# Chitosan and carboxymethyl chitosan nanocarriers enhance mango seed extract stability and antimicrobial activity to improve strawberry postharvest quality

**DOI:** 10.1038/s41598-025-16756-1

**Published:** 2025-08-26

**Authors:** Eman S. El-Ashaal, Hisham A. Elshoky, Nayera M. El-Sayed, Ebtehal A. El-Kholany

**Affiliations:** 1 Nanotechnology and Advanced Material Central Lab, Agricultural Research Center, Giza, 12619 Egypt; 2 Regional Center for Food and Feed, Agricultural Research Center, Giza, 12619 Egypt; 3 Physics Department, Faculty of Science, Mansoura University, Mansoura, 35516 Egypt; 4 Special Food and Nutrition Department, Food Technology Research Institute, Agricultural Research Center, Giza, 12619 Egypt

**Keywords:** Chitosan, Carboxymethyl chitosan, Mango seed extract, Strawberry, Biopolymers edible coatings, Biological techniques, Biotechnology, Nanoscience and technology

## Abstract

Strawberries are highly perishable fruits due to moisture loss, intense metabolic activity, and microbial contamination, leading to rapid quality deterioration during storage. In this study, mango seed extract (MSE), a natural antioxidant source, was loaded with chitosan (CS) and carboxymethyl chitosan (CCS) nanoparticles to develop edible coatings for postharvest quality enhancement. Nanoparticles were characterized by dynamic light scattering (DLS) to determine particle size and zeta potential, revealing stable formulations with average sizes of 68.1 nm (CS LM) and 91.3 nm (CCS LM). Antioxidant capacity was assessed using ABTS and DPPH radical scavenging assays, where CCS LM exhibited superior activity (> 92%, *p* < 0.05) compared to free MSE and CS LM. Antimicrobial properties were evaluated by microbial inhibition tests against *Staphylococcus aureus*, *Escherichia coli*, and *Candida albicans*, showing significant inhibition rates at 800 µg/mL. Storage performance was assessed over 21 days at 2 °C by monitoring weight loss, firmness, color preservation, pH, titratable acidity, and microbial counts. Results demonstrated that CCS LM-coated strawberries had minimal weight loss (5.7%, *p* < 0.05), retained firmness, and showed reduced bacterial (1.2 log₁₀ CFU/g) and fungal counts (1.3 log₁₀ CFU/g) compared to uncoated fruits. These findings suggest that MSE-loaded CCS nanoparticles, through their antioxidant and antimicrobial functions, offer a promising sustainable strategy to improve the postharvest quality and storage performance of strawberries.

## Introduction

Strawberries are a widely consumed fruit known for their appealing taste, rich antioxidant content, and numerous health benefits, such as vitamins, minerals, and polyphenolic compounds^1,2^. However, strawberries are also among the most perishable fruits due to their high moisture content, soft texture, and vulnerability to microbial contamination. These factors significantly limit their post-harvest shelf life, leading to rapid quality deterioration during storage, particularly when subjected to mechanical damage or suboptimal conditions^3,4^. Moreover, strawberries are highly susceptible to dehydration, color loss, softening, and microbial spoilage from a range of bacteria, molds, and yeast. Consequently, ensuring quality and extending the shelf life of strawberries has become a critical area of research in food science, especially for markets where long-distance transportation and extended storage are necessary^5^.

To address the limitations of traditional preservation methods, recent studies have explored the use of edible coatings as a sustainable and effective alternative for post-harvest preservation. Edible coatings are thin layers applied to the surface of fruits to create a semi-permeable barrier that can slow respiration rates, reduce moisture loss, and delay spoilage. Unlike synthetic packaging, edible coatings are typically biodegradable and safe for consumption, and they can be fortified with natural antimicrobial and antioxidant agents to further enhance their protective effects^6,7^. In recent years, biopolymer-based edible coatings, such as those made from chitosan (CS) and carboxymethyl chitosan (CCS), have shown considerable promise in extending the shelf life of fresh products. Chitosan (CS), a polysaccharide derived from chitin, is particularly valued in food preservation due to its biocompatibility, biodegradability, and intrinsic antimicrobial properties^8^. It can form a film that acts as a barrier to oxygen, moisture, and other gases, which helps maintain the structural integrity and quality of the coated produce, due to its low solubility in neutral and alkaline conditions, which limits its use. Carboxymethyl chitosan (CCS), a modified structure of CS, enhances solubility, film-forming, and increased flexibility, which increases its potential use in advanced edible coatings^9^.

In parallel with these developments, active food packaging technologies have rapidly evolved to address consumer demands for fresher, safer, and longer-lasting food products. Active packaging materials are designed not only to passively protect food but also to actively interact with the food environment to inhibit microbial growth, reduce oxidation, and extend shelf life. Recent innovations include the use of biomimetic materials that mimic natural protective mechanisms, self-healing materials that repair micro-damage during storage, and nanomaterials that provide enhanced barrier and functional properties^10,11^. In particular, nanomaterials offer increased surface area and reactivity, improving the delivery and stability of active compounds such as antioxidants and antimicrobials^12^.

Plant-extract-based antibacterial materials are also gaining popularity, offering a natural, safe, and sustainable means of enhancing food preservation without relying on synthetic additives^13,14^. These active packaging strategies align with the broader trends toward clean-label, eco-friendly technologies in the food industry^15^.

Nanotechnology has enhanced bioactive compound evaluation, providing advantages over conventional methods^16^. Nanomaterials offer increased surface area and contact with food matrices. Thus, nanoscale bioactive compounds significantly benefit the food, nutraceutical, pharmaceutical, and agricultural industries by improving their stability, controlled release, and resistance to degradation factors^17^. Using edible polymers and natural additives, such as plant extracts rich in bioactive compounds, in these coatings extends the shelf life and enhances the quality of fresh fruits and vegetables, providing additional protection and preservation^18^. Commonly used active packaging materials include carbohydrate polymers such as CS and carboxymethyl chitosan (CCS), known for their biodegradability, biocompatibility, film-forming capability, and edibility. They can be used as packaging films or directly applied as coatings on food products^19^. Strawberries have a short shelf life due to their high metabolism, water content, and susceptibility to damage^19,20^. For instance, coatings combining CS with sea buckthorn or grapeseed oils inhibited strawberry microbial growth and physicochemical changes during 7 days of cold storage, outperforming uncoated controls^20^.

Global agro-industrial processes yield substantial quantities of raw materials and energy, primarily for human and animal consumption^21^. The fruit and vegetable processing industries generate significant waste, including peels, pomace, and seed fractions. These waste materials contain valuable bioactive compounds with recognized health benefits, exhibiting anticancer, antiviral, antioxidant, and antitumor activity^22,23^. Thus, efficient strategies are needed to repurpose these residues for higher-value products to enhance sustainability. Mango (*Mangifera indica L.*) is a highly valued exotic fruit that generates substantial byproducts, including peels and seeds (35–60% of the whole fruit)^16^. Mango seeds (10–25% of the fruit) contain bioactive compounds, such as phenolic compounds and flavonoids, known for their antioxidant, antimicrobial, and anti-inflammatory properties. Techniques such as solvent extraction can effectively recover these compounds from mango seeds^17,24^. However, the challenge lies in the sensitivity of bioactive compounds to factors such as temperature, light, and degradation, which limits their efficacy and reduces their effectiveness. The limited solubility of these compounds in water and oil further impedes their practical use in food^18^. The integration of these extracts into advanced packaging systems, especially using nanotechnology, offers promising solutions to enhance their stability and functionality during food preservation. Further, the utilization of nanotechnology to stabilize MSE in edible coatings has not yet been fully explored.

The hypothesis of this study was that the MSE-loaded CS and CCS nanoparticle coatings would significantly reduce weight loss, maintain firmness and color, slow down changes in titratable acidity and pH, and inhibit microbial growth more effectively than uncoated controls or coatings with MSE. By combining the antimicrobial and antioxidant properties of MSE with the film-forming and protective capabilities of CS and CCS, the study aimed to demonstrate the potential of these coatings as sustainable, bioactive solutions for post-harvest preservation. This approach could offer an eco-friendly alternative to synthetic preservatives and packaging materials, aligning with the increasing consumer demand for natural and minimally processed food products.

This study explores the integration of MSE into chitosan and carboxymethyl chitosan nanoparticles as a novel approach to provide a sustainable solution for enhancing the stability of MSE, as well as, creating edible coatings for strawberry preservation. By evaluating physicochemical stability, antioxidant activity, antimicrobial efficacy, and overall preservation of fruit quality, the research aims to provide comprehensive insights into the mechanisms and benefits of using biopolymeric nanoparticle-based coatings for extending the shelf life of perishable fruits. This interdisciplinary strategy offers a forward-looking perspective on how active packaging systems, particularly those leveraging nanotechnology and plant-derived bioactives, can revolutionize the future of food preservation technologies.

Moreover, it contributes to global efforts in reducing food waste, promoting sustainability, and meeting the rising consumer expectations for safe, natural, and eco-friendly food products.

## Materials and methods

### Materials

#### ***Chemicals*** 

Folin-Ciocalteu reagent, 2,2-diphenyl-1-picrylhydrazyl (DPPH), 2,2’-azino-bis(3-ethylbenzothiazoline-6-sulfonic acid (ABTS), and low-molecular-weight chitosan (MW: 112,000 kDa, degree of deacetylation: ≥75%) were obtained from Acros Organics (USA). Carboxymethyl chitosan (CCS) was obtained from the Nanotechnology and Advanced Material Central Lab (NAMCL), Agricultural Research Center, Egypt. Sodium tripolyphosphate (TPP), gallic acid standard, and glacial acetic acid were obtained from Sigma‒Aldrich (USA) and Merck (Germany). All chemicals were of analytical grade and used without further purification.

#### Raw materials

Mango fruits (*Mangifera indica* L., ‘Owais’ cultivar) were procured from local farms in Giza, Egypt. Seeds were manually extracted using a sterilized stainless-steel knife, thoroughly washed with distilled water, and dried in a hot air oven at 45 ± 2 °C until a constant weight was achieved. The dried seeds were then finely ground using a hammer mill (model SR-300, Brightsail Machinery, China) to obtain a uniform powder for subsequent extraction of bioactive compounds^25^. Fresh strawberries (*Fragaria × ananassa* Duch., ‘Festival’ variety) at approximately 75% ripeness (characterized by uniform red coloration and consistent size) were harvested from Giza farms. After collection, strawberries were washed with distilled water, drained for 10 min at room temperature, and randomly divided into four experimental Owais.

### Preparation of mango seed extract

Mango seeds (*Mangifera indica* L., cultivar ‘Owais’) were collected from ripe fruits obtained from a local market (Giza, Egypt). The seeds were manually separated, washed with distilled water to remove adhering pulp, and dried at 40 °C in a hot air oven until reaching constant weight. The dried seeds were then ground into a fine powder using a laboratory mill and sieved to obtain a uniform particle size. Mango seed powder (10 g) was subjected to maceration extraction using a magnetic stirrer operating at 3000 rpm. The powder was extracted three times with 100 ml of 70% (v/v) ethanol at 25 °C for 48 h in the dark. After each extraction, Whatman No. 1 filter paper was used to filter and pool the filtrates. The mixed extracts were concentrated at 40 °C under decreased pressure in a rotary evaporator (IKA RV 10 basic, UK) to remove ethanol. The concentrated extract was freeze-dried using a lyophilizer (Snijders Scientific, Type 2040, Netherlands) to obtain a dry powder. The MSE powder was stored at 4 ± 1 °C in airtight, light-protected containers until further analysis to preserve its bioactive compound content^26^.

### Total phenolic (TPC) and flavonoid content (TFC)

#### Total phenolic content (TPC)

The TPC was quantified using the Folin–Ciocalteu colorimetric method as per Wolfe et al.^27^, with slight modifications. Briefly, 0.5 ml of mango seed extract (10 mg/ml) was mixed with 2.5 ml of 10% Folin–Ciocalteu reagent and allowed to react for 5 min. Subsequently, 2 ml of sodium carbonate solution (75 g/l) was added. The mixture was incubated at room temperature in the dark for 30 min, and the absorbance was measured at 760 nm using a UV-Vis spectrophotometer (Shimadzu UV-1800, Japan). Gallic acid was used to prepare the calibration curve (concentration range: 20–200 µg/ml). The results were expressed as milligrams of gallic acid equivalent (mg GAE) per 100 g dry weight (DW) of sample.

#### Total flavonoid content (TFC)

TFC was measured according to the aluminum chloride colorimetric method described by Zhishen et al.^28^. A volume of 0.5 ml of extract was mixed with 2 ml of distilled water, followed by the addition of 0.15 ml of 5% NaNO₂ solution. After 6 min, 0.15 ml of 10% AlCl₃ solution was added, and the reaction mixture was incubated for another 6 min. Then, 2 ml of 4% NaOH was added, and the final volume was adjusted to 5 ml with distilled water. The solution was incubated at room temperature for 15 min, and absorbance was measured at 510 nm. Quercetin was used as the standard, and the results were expressed as mg quercetin equivalent (mg QE) per gram of sample.

### Preparation of chitosan (CS) and carboxymethyl chitosan (CCS) nanoparticles

CS and CCS nanoparticles (NPs) were synthesized using the ionic gelation method, adapted from previous protocols^8,29–32^. Specifically, 100 mg of CS or CCS was dissolved in 94 ml of deionized water containing 1 ml of glacial acetic acid (1% v/v) and stirred overnight at room temperature until a clear solution was obtained. Sodium tripolyphosphate (TPP; 33 mg) was separately dissolved in 5 ml of deionized water. The TPP solution was added dropwise to the CS or CCS solution under continuous stirring at 800 rpm for 30 min to promote crosslinking and nanoparticle formation. The resulting suspension was stored at 4 °C until further use.

### Preparation of CS and CCS NPs conjugated with mango seed extract

To formulate mango-loaded nanoparticles (CS LM and CCS LM), 25 ml of mango seed extract solution (dissolved in double-distilled water at a concentration of 10 mg/ml) was mixed with 25 ml of freshly prepared CS or CCS nanoparticles suspension (1:1 v/v ratio). The 1:1 ratio was selected based on preliminary optimization experiments, where equal volumes ensured efficient encapsulation without nanoparticle aggregation or phase separation. The mixture was stirred continuously at 500 rpm using a magnetic stirrer overnight (~ 16 h) at room temperature (25 ± 1 °C) under dark conditions to protect the bioactive compounds from light-induced degradation. These re-dispersed nanoparticle suspensions were subsequently used for further characterization, including particle size, zeta potential, antioxidant activity, and antimicrobial efficacy analyses.

### Structural characterization of CS and CCS NPs conjugates with mango seed extract

CS and CCS NPs conjugates with mango extract (CS LM and CCS LM) were characterized using different techniques. The particle size distribution and zeta potential of the nanoparticles were determined using a Malvern Zetasizer (Nano ZS90, UK). The samples were diluted in deionized water to ensure the measurements fell within the optimal scattering intensity range of the instrument. The average particle size and polydispersity index (PDI) were recorded. Zeta potential measurements were performed to assess the surface charge of the nanoparticles, which is an important parameter influencing the stability of nanoparticle suspensions. The functional groups present in the mango seed extract, chitosan, carboxymethyl chitosan, and the nanoparticles were analyzed by Fourier Transform Infrared Spectroscopy (FTIR, JASCO FTIR 6100, USA). The samples were lyophilized and pressed into KBr pellets. FTIR spectra were recorded in the 4000–500 cm⁻¹ range with a resolution of 4 cm⁻¹. The spectra were analyzed to identify characteristic peaks corresponding to the functional groups of chitosan, carboxymethyl chitosan, and bioactive components from the mango seed extract, such as polyphenols.

### Stability study of CCS LM, CS LM, and lyophilized mango seed extract (MSE)

The stability of the total phenolic content in the samples loaded with chitosan (CS LM) and carboxymethyl chitosan (CCS LM) was assessed using the Folin–Ciocalteu method, following a previously described protocol^33^. Stability was monitored over 60 days, with sampling at intervals of 0, 7, 14, 30, 45, and 60 days. The samples were stored under two different temperature conditions: refrigerated (4 °C) and ambient (~ 25 °C) temperatures, to simulate different storage environments. For comparative analysis, lyophilized mango seed extract (MSE) was used as the control. The total phenolic content was determined at each time point to evaluate the retention of bioactive compounds and assess the effect of storage conditions on the stability of the encapsulated extract.

### Antioxidant activity using ABTS and DPPH methods

#### ABTS assay

The antioxidant activity of the samples was evaluated using the ABTS (2,2’-azino-bis (3-ethylbenzothiazoline-6-sulphonic acid)) radical cation decolorization assay, based on a previously established method^34^. The ABTS radical was generated by reacting ABTS (7 mM) with ammonium persulfate (2.45 mM) and allowing the mixture to incubate in the dark at room temperature for 16 h. After incubation, the ABTS radical solution was diluted with ethanol to achieve an absorbance of 0.7 ± 0.02 at 734 nm. A 20 µl aliquot was added to 980 µl of ABTS solution to homogenize the sample. Incubation at room temperature for 6 min and measured at 734 nm. The ABTS radical scavenging activity was calculated using the following formula:

$${\rm ABTS\: scavenging\: activity}\:(\%)= \frac{\boldsymbol{A}\left(0\right)\: -\:\mathbf{A}\left(1\right)}{\mathbf{A}\left(0\right)\:} \times 100$$

**where**:

A0 is the absorbance of the ABTS solution at time zero (without the sample),

A1 is the absorbance after adding the sample.

#### DPPH assay

The antioxidant activity of the samples was also evaluated using the DPPH (2,2-diphenyl-1-picryl-hydrazyl) method^35^. Briefly, 100 µl of the sample extract was added to 4 ml of DPPH solution (6 × 10⁻⁵ M in methanol). The mixture was vortexed and incubated in the dark at room temperature for 30 min. The absorbance of the mixture was recorded at 517 nm. The percentage of DPPH radical inhibition was calculated using the following formula:

$${\rm Inhibition (\%) }= \:\frac{{\boldsymbol{A}}_{\boldsymbol{C}}\left(0\right)\: -\:{\boldsymbol{A}}_{\boldsymbol{A}}\left(\mathbf{t}\right)}{{\boldsymbol{A}}_{\boldsymbol{C}}\left(0\right)\:} \times 100$$

Where Ac (0) is the absorbance of the control at time 0 min, A_A_ (t) is the absorbance of the antioxidant at 30 min.

### Antimicrobial activity determination by the broth Dilution method

Antimicrobial activity was evaluated using the broth dilution method. Different concentrations of the test materials (200, 400, 600, and 800 µg/ml) were added to 500 µl tubes containing Muller-Hinton broth, which was then inoculated with three different microorganisms: *Escherichia coli* (initial count: 6.5 log_10_ CFU/g), *Staphylococcus aureus* (6.3 log_10_ CFU/g), and *Candida albicans* (5.9 log_10_ CFU/g). The inoculated broth was incubated under the following conditions: bacterial strains were incubated at 37 °C for 24 h, and the fungal strain was incubated at 30 °C for 48 h. The growth of the microorganisms was monitored by measuring the optical density at 600 nm. The OD measurements were used to monitor turbidity changes, and antimicrobial activity was expressed as a percentage of inhibition compared to controls. The results were expressed as the percentage of growth inhibition against each strain^36^:

$${\rm Percentage \:of \:growth\: Inhibition (\%)} \:=\: \:\frac{\mathrm{O}\mathrm{D}\:\mathrm{c}\mathrm{o}\mathrm{n}\mathrm{t}\mathrm{r}\mathrm{o}\mathrm{l}\text{}\:-\mathrm{O}\mathrm{D}\:\mathrm{s}\mathrm{a}\mathrm{m}\mathrm{p}\mathrm{l}\mathrm{e}}{\mathrm{O}\mathrm{D}\:\mathrm{c}\mathrm{o}\mathrm{n}\mathrm{t}\mathrm{r}\mathrm{o}\mathrm{l}}\times\:100$$

**OD control​** is the optical density of the control sample (without the tested material).

**OD sample​** is the optical density of the sample containing the tested material.

### Live/Dead staining and confocal microscopy

confocal microscopy technique employing acridine orange (AO) and propidium iodide (PI) stains was used for live/dead staining assays^8,31,37^. AO, a fluorescent dye, enters living bacterial cells, and produces a green fluorescence signal. Dead cells were differentiated using PI, which penetrates only damaged cell membranes, and appears red fluorescence signal. Confocal microscopy analysis was performed using an LSM 710 Zeiss confocal microscopy system with a 40× objective. Image analysis with ZEN 3.2 Pro software was used to quantify living and dead bacteria, allowing to determine the percentage of each bacteria. Representative images were acquired to visualize the results.

### Coating application

Fresh strawberries (*Fragaria × ananassa*) were disinfected by soaking them in a 1% sodium hypochlorite (NaClO) solution for 5 min, followed by thorough rinsing with distilled water to remove any residual chlorine. The strawberries were air-dried under sterile conditions to ensure complete removal of excess water. For coating application, strawberries were individually dipped into one of the following coatings: 800 µg/ml mango seed extract (MSE), chitosan-loaded mango seed extract (CS LM), or carboxymethyl chitosan-loaded mango seed extract (CCS LM) conjugates. Each batch of strawberries was immersed in the coating solution for 60 s, ensuring complete coverage. After coating, excess solution was drained off, and the strawberries were air-dried at 25 °C for 2 h. The coated strawberries were placed in polypropylene (PP) cartons and refrigerated at 2 °C with 95% RH to simulate typical refrigerated storage conditions. This temperature was chosen to reflect commonly used low-temperature storage conditions for strawberries, as refrigeration at this temperature reduces metabolic activity and helps extend the shelf life of the fruit. The strawberries were evaluated for quality, including weight loss, firmness, color, and microbial load, at regular intervals (0, 3, 7, 10, 14, and 21 days) during the storage period.

### Quality parameters

During storage, several postharvest quality parameters of strawberries were monitored to assess the effectiveness of the applied coatings. These parameters included weight loss, firmness, color, pH, titratable acidity, and microbial counts. The following quality parameters were evaluated during the storage of the coated and uncoated strawberries:

#### Weight loss (%)

The weight loss of strawberries during storage was calculated using the following equation:

 

$${\rm Weight \:loss (\%)} = \:\frac{{\boldsymbol{W}}_{0}-{\boldsymbol{W}}_{\boldsymbol{i}}}{{\boldsymbol{W}}_{0}} \times 100$$

Where W_0_is the initial weight and W_i_ ​ is the final weight at each time point.

#### Firmness

Strawberry firmness was measured using a universal testing machine (Cometech, B Type). A force was applied to the strawberry, and the puncture test was conducted at a speed of 1 mm/s to a depth of 25% using an Aluminum 25 diameter cylindrical probe. The firmness was calculated from the force-time curve obtained during the test.

#### Titratable acidity (TA) and total soluble solids (TSS)

For TA and TSS measurement, 10 g of strawberry was homogenized in 100 ml of water and centrifuged at 4000 rpm for 10 min. The supernatant was then collected and used for further analysis. TA was determined by titrating the supernatant to pH 8.1 using 0.1 M NaOH and was expressed as citric acid content^38^. TSS was measured using a refractometer.

#### pH

The pH of the strawberry extract was measured using a pH meter (HI 9126, Hanna Instruments Inc., Romania).

#### Color measurement

The color of the strawberries was assessed using a Minolta Chroma Meter CR-300 (EC Minolta, Japan). The color parameters L*, a*, and b* were recorded, where L* represents lightness (0 = black, 100 = white), a* indicates redness, and b* represents yellowness^39^.

#### Anthocyanin content

Five grams of strawberry was homogenized in 40 ml of absolute ethanol containing 1.5 M HCl. After filtering the mixture, the volume was adjusted to 100 ml with distilled water, and the absorbance was measured at 535 nm using a spectrophotometer to determine the anthocyanin content^40^.

In addition to these parameters, all coated and uncoated strawberries were analyzed for microbial contamination. Aerobic plate counts, presence of psychrophilic bacteria, and the presence of yeast and mold were evaluated according to the methods of the American Public Health Association^41^.

### Statistical analysis

The statistical analysis employed one-way ANOVA with a significance level of 0.1234 using CoStat (ver. 6.400)^42^. The data were treated as a complete randomization design, and the least significant difference (LSD) test was applied to assess the significance among means of different samples. A threshold of *p* < 0.05 was set to determine statistical significance. A significance threshold of ^ns^*p* < 0.1234 was applied, and significance levels were denoted in graphs as follows: ^*^p < 0.0332, ^**^p < 0.0021, ^***^p < 0.0002, and ^****^p < 0.0001 for easy interpretation of statistical findings.

## Results and discussion

### Extract yield, total phenolic and flavonoid content of mango seed powder, and its ethanolic extract

The results of the quantitative screening of the phytochemical constituents revealed that the ethanolic extract had higher levels of phenolic and flavonoid contents (412.21 ± 4.17 mg GAE/g and 69.82 ± 1.55 mg QC/g, respectively), which were significantly greater than those of the powder (81.78 ± 1.83 mg GAE/g and 14.09 ± 0.32 mg QC/g, respectively; *p* < 0.1234). These results are in line with those of Bernal-Mercado et al.^25^., who reported that the total phenolic and flavonoid contents of mango seed ethanolic extracts were 484.42 mg GAE/g and 86.59 mg QE/g, respectively. Mango seed extract (MSE) was obtained using a hydroethanolic solution (70% ethanol and 30% water) as a solvent with an extraction yield of 8.52%. The phytochemical content and high extraction yield obtained with the mixed solvent system (70% ethanol and 30% water) are due to the optimum polarity of hydroethanol, which enhances the solubility and release of phenolic compounds from the plant matrix, as pure solvents tend to be less effective^43^. The obtained yield was close to that of El-Kady et al.^44^., who noted that the extraction yield of mango seed kernel powder was 9.43 g/100 g.

### Structural characterization of the prepared materials

**Fig. 1:**
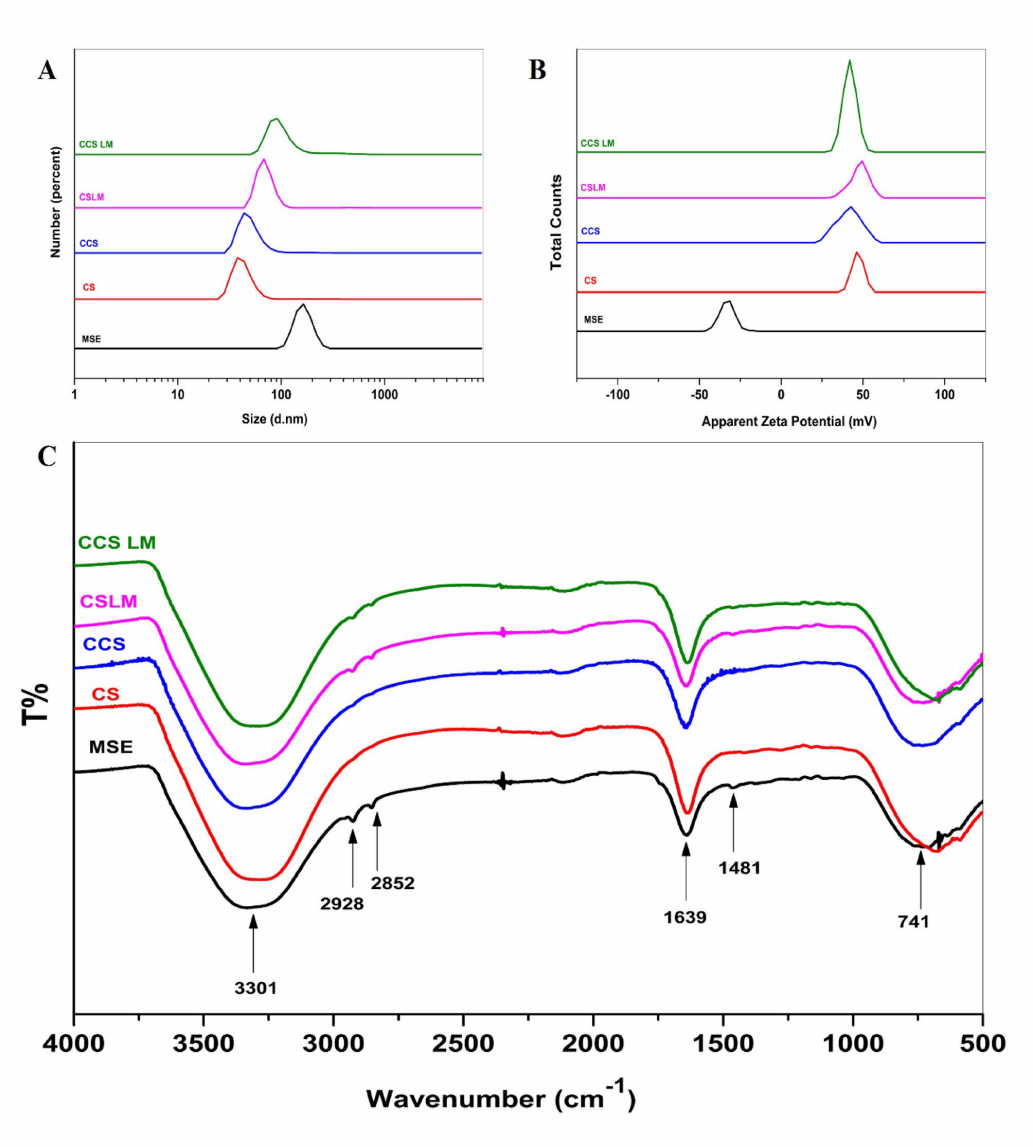
*Characterization of nanoparticles.* *(a) Particle size distributions of MSE*,* CS NPs*,* CCS NPs*,* and mango-loaded CS and CCS NPs (CS LM and CCS LM). (b) Zeta potentials of the same formulations indicating surface charge and colloidal stability. (c) FTIR spectra confirming functional group interactions between MSE and CS/CCS matrices.*

When mango seed extracts (MSE) are loaded with CS NPs and CCS NPs, DLS can provide valuable information about the size distribution (the hydrodynamic diameter) and surface charge (zeta potential) of the prepared materials. In Fig. 1**(a)**, each peak represents the particle size distribution of a different sample: MSE, CS NPs, CCS NPs, CS LM (MSE loaded with CS NPs), and CCS LM (MSE loaded with CCS NPs). The sizes of the prepared materials were found to follow a normal distribution curve. The size of the MSE was 164.2 ± 31.06 d.nm, with a polydispersity index (PDI) of 0.858 (Fig. 1**(a)**, Table 1). The size distribution of the prepared CS and CCS NPs was also measured, which were 37.8 ± 9.38 d.nm with 0.262 PDI and 43.8 ± 23.63 d.nm with 0.436 PDI (Fig. 1(a), Table 2). The relatively low PDI values for CS and CCS NPs indicate that the nanoparticle suspensions were monodisperse and homogeneous.

MSE was measured again after loading with CS and CCS NPs and is shown in the same Fig. 1**and** Table 1. MSE particle sizes decreased after loading with CS and CCS NPs, which had particle sizes of 68.1 ± 12.41 and 91.3 ± 30.05 d.nm, respectively. This reduction in size upon loading suggests successful interaction and encapsulation of MSE within the nanoparticle matrices. Compared to MSE, the CS LM and CCS LM PDI reduced to 0.506 and 0.574, showing homogeneity. According to El-Sayed et al.^45^, nanoparticles with zeta potentials greater than + 30 mV or less than − 30 mV generally exhibit good colloidal stability due to sufficient electrostatic repulsion.

The zeta potential of MSE after loading with CS NPs and CCS NPs was also measured. As shown in Table 2, the ZP of the CS LM and CCS LM were + 47.8 ± 5.62 mV and + 41.9 ± 4.06 mV, respectively. The positive surface charge after loading indicates successful surface modification and encapsulation. The produced CS LM and CCS LM have desirable ZP values, i.e., more than the + 30 mV required for stable formulations. In the present study, CS LM and CCS LM had zeta potential values confirming their excellent dispersibility and stability, making them promising candidates for bioactive delivery applications. Furthermore, the size and zeta potential results in Fig. 1b have been discussed in relation to previous studies to emphasize the successful fabrication and stability of the nanoparticles.

**Table 1:** *The hydrodynamic diameter (nm) distribution*,* polydispersity index*,* and zeta potentials of MSE*,* CS*,* CCS*,* and MSE after loading with CS and CCS determined via DLS.*

NPs	Particle size (nm)	PDI	Zeta potential (mV)
MSE	164.2 ± 31.06	0.858	−33 ± 4.67
CS NPs	37.8 ± 9.38	0.262	+ 46.8 ± 3.53
CCS NPs	43.8 ± 23.63	0.436	+ 41.3 ± 7.51
CS LM	68.1 ± 12.41	0.506	+ 47.8 ± 5.62
CCS LM	91.3 ± 30.05	0.574	+ 41.9 ± 4.06

FTIR is an operative technique for the fast identification of MSE and its loaded extract (CS LM and CCS LM) and for determining the interface between the extract and the prepared nanoparticles. Figure 1c shows the FTIR spectra of raw (MSE) and treated mango seed extracts at 4000–500 cm^−1^ resolution. The MSE spectrum revealed peaks at 3301 cm^−1^ for alcohol O-H stretching, 2910 and 2852 cm^−1^ for alkane C-H stretching, 1639 cm^−1^ for conjugated alkene C = C stretching, and 1481 and 741 cm^−1^ for alkane methyl group C-H bending. The spectra of CS and CCS NPs showed different peaks at 3291 and 3306 cm^−1^, respectively. These peaks are caused by O-H stretching vibrations.

Additionally, there were also Peaks at 1643 and 1636 cm^−1^ may indicate stretching vibrations of symmetric and asymmetric carbonyl groups, possibly from adsorbed acetic acid. In conclusion, the peaks at 667 and 731 cm^−1^ correspond to the C-H bending vibrations of alkane groups. Additional peaks were seen in the CS LM and CCS LM. The new peaks are slightly shifted versions of the MSE peaks, shifting to 2929 cm⁻¹ and 2848 cm⁻¹. Additional shifts were seen in C = C and C-H group peaks. Indeed, loading CS and CCS NPs onto MSE caused a dramatic shift in existing peaks and the development of new ones. The nanoparticle-MSE connection is suggested by this finding.

### Determination of the antioxidant activity

The ABTS and DPPH assays measure radical scavenging activity through different mechanisms, specifically electron transfer (ET) for ABTS and hydrogen transfer (HT) for DPPH. The antioxidant activity of the mango seed extracts (MSE) and mango seeds extract loaded with chitosan nanoparticles (CS LM) and carboxymethyl chitosan nanoparticles (CCS LM) was determined (Table 2). The highest scavenging activity (%) using ABTS and DPPH was observed for CCS LM (95.00 ± 0.28% and 92.55 ± 0.19%, respectively), followed by CS LM (91.02 ± 0.49% and 90.17 ± 0.14%, respectively). Additionally, CCS LM exhibited the highest antioxidant activity (IC_50_ as Trolox equivalents) with 7.11 µg Trolox/ml for DPPH and 4.65 µg Trolox/ml for ABTS followed by CS LM compared to MSE.

During ionic gelation, the amino groups of chitosan and carboxymethyl chitosan cross-link with phenolic compounds in mango seed extract, enhancing the antioxidant activity of the loaded mango seed extract with CCS and CS nanoparticles. The volatile nature of mango seed extract can lead to evaporation, but the loading technique helps preserve phenolic compounds by reducing the evaporation rate, thereby suppressing free radicals^46^. Previous studies have shown that the antioxidant activity of aqueous grape extract loaded in chitosan-TPP nanoparticles is greater than that of chitosan. Our findings were consistent with those of previous investigations^47^.

The highest antioxidant activity of CCS LM, determined by ABTS and DPPH, could be attributed to the enhanced interactions between carboxymethyl chitosan (CCS) and the phenolic compounds in mango seed extract. CCS has carboxymethyl groups (–CH_2_–COOH), which make it more soluble in water and form a more homogeneous matrix with phenolic compounds, which improves their accessibility and stability, leading to improved antioxidant activity. Furthermore, during the ionic gelation process, the amino groups of CCS cross-link with phenolic compounds, enhancing loading and retention. Hydrogen bonding and electrostatic interactions are increased by carboxymethyl groups, stabilizing the structure and preventing degradation. Moreover, CCS also imparts antioxidant activity to the carboxymethyl groups, which serve as radical scavengers. Therefore, the overall antioxidant activity of CCS and phenolic compounds is more significant than the individual form. On the other hand, CS LM exhibited lower antioxidant activity due to the decreased solubility and loading efficiency of chitosan. MSE alone possessed the lowest antioxidant activity due to low stability and fast release^48–50^.

**Table 2:** *Antioxidant activity of MSE*,* CS LM*,* and CCS LM.*

AOA Samples	DPPH		ABTS	
	%	IC_50_ µg Trolox/ml	%	IC_50_ µg Trolox/ml
**MSE**	87.71^c^ ± 0.59	9.84	89.25^c^ ± 00.75	7.95
**CS LM**	90.17^b^ ± 0.14	8.41	91.02^b^ ± 0.49	5.85
**CCS LM**	92.55^a^ ± 0.19	7.11	95.00^a^ ± 0.28	4.65
**LSD ≥ 0.05**	0.73		1.08	

### Stability study of mango seed extract loaded with CS and CCS and lyophilized mango seed extract (MSE)

**Fig. 2:**
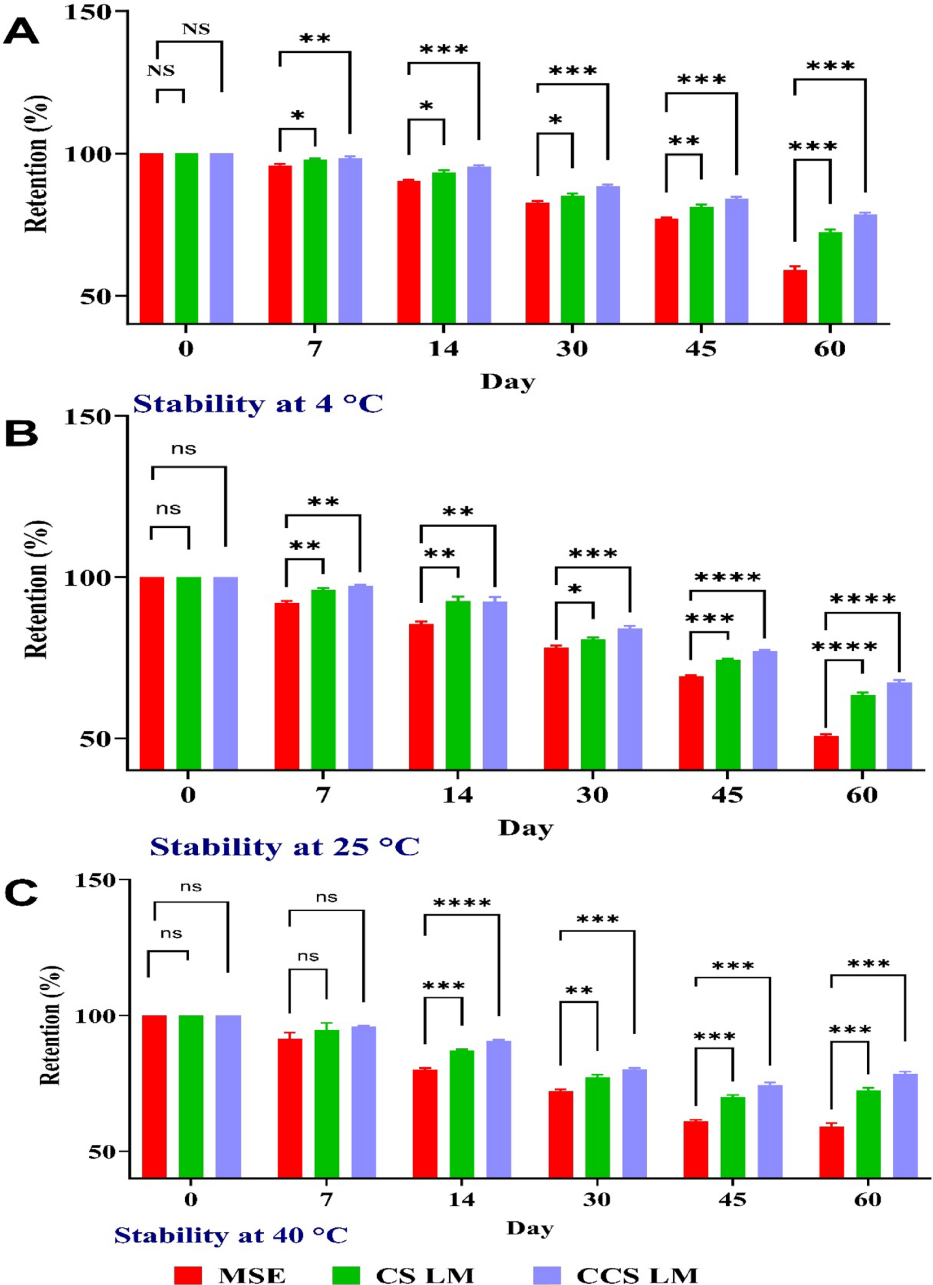
*Stability of total phenolic content (TPC) over 60 days.* *Retention of TPC in MSE*,* CS LM*,* and CCS LM at (A) 4 °C*,* (B) 25 °C*, and (C) 40 °C. Significance: CCS LM showed significantly higher retention compared to MSE at all time points (*p* < 0.1234).

Mango seed extract loaded with CS NPs and CCSNPs (CS LM and CCS LM) as well as mango seed extract (MSE) were applied on the day of manufacture and the 7th, 14th, 30th, 45th, and 60th days at 4, 25, and 40 °C (*P* < 0.1234 at all the studied time). The results are displayed in Fig. 2. Compared with MSE alone, loading mango seed extract (MSE) with chitosan (CS) and carboxymethyl chitosan (CCS) nanoparticles resulted in significantly greater phenolic compound retention at all tested temperatures during the 60-day experiment. CCS LM and CS LM exhibit a slight initial decrease, followed by a more significant decline between the thirtieth and sixth days. At the end of the experiment, CCS LM retained 78.50% at 4 °C, 67.35% at 25 °C, and 60% at 40 °C, while CS LM retained 72.44%, 63.47%, and 54.28%, respectively. In contrast, MSE experienced substantial degradation, with a loss of 59.0% at 4 °C, 50.59% at 25 °C, and 41.87% at 40 °C after 60 days (*P* < 0.1234). On the other hand, the most degraded samples were observed at 40 °C, demonstrating the great sensitivity of phenolic compounds to temperature. Compared with MSE alone, loading mango extract with CS and CCS nanoparticles provided better protection against phenolics. These results agreed with those of^51^, who reported that the microencapsulation of taperebá peel significantly protected the phenolics in the extract, with a retention of ~ 60%. In comparison, the lyophilized peel extract (LPE) showed a retention of ~ 35%.

### Antimicrobial activity determination by turbidity

The antimicrobial activity of MSE, CS LM, and CCS LM was evaluated using the turbidity method at various concentrations (200, 400, 600, and 800 µg/ml), and the results are expressed as inhibition percentages (Table 3). All the samples exhibited inhibitory effects on the tested bacterial and fungal strains (*E. coli*, *S. aureus*, and *C. albicans*), increasing inhibition at higher concentrations. CCS LM demonstrated the highest inhibitory activity against *S. aureus* (81.51%) and *E. coli* (75.29%), followed by CS LM (77.46 and 71.27%, respectively), compared to MSE (64.99 and 57.66%, respectively) at 800 µg/ml. Additionally, compared with MSE, CCS LM had the most significant inhibitory effect on *C. albicans* (70.99%), followed by CS LM (66.29%). MSE exhibited the lowest inhibitory activity against all strains at different concentrations. These results agreed with^52^, indicating the effective antimicrobial activity of chitosan loaded with *H. perforatum* and *S. officinalis* extracts against *S. aureus*, *E. coli*, and *P. aeruginosa*.

**Table 3:** *Inhibition percentage (%) of MSE*,* CS LM*,* and CCS LM against some pathogenic bacteria and fungi according to optical density.*

Pathogenic Concentration(µg/ml)		Gram-Negative	Gram-Positive	Fungi
		*E.coli*	*S.aureus*	*C.albicans*
**MSE**	**200**	41.81^i^ ± 1.43	45.51^h^ ± 3.44	39.80^g^ ± 2.05
	**400**	46.16^h^ ± 1.23	49.22^g^ ± 1.96	44.29^f^ ± 1.76
	**600**	51.69^g^ ± 0.81	56.53^f^ ± 2.27	46.15^f^ ± 0.97
	**800**	57.66^ef^ ± 2.01	64.99^d^ ± 1.15	50.57^e^ ± 1.13
**CS LM**	**200**	50.26^g^ ± 1.64	54.43^f^ ± 2.30	44.82^f^ ± 1.77
	**400**	58.33^e^ ± 1.30	63.41d^e^ ± 1.98	53.85^d^ ± 1.72
	**600**	67.56^c^ ± 1.00	72.16^c^ ± 1.91	61.37^c^ ± 1.49
	**800**	71.27^b^ ± 1.29	77.46^b^ ± 1.32	66.29^b^ ± 1.22
**CCS LM**	**200**	55.48^f^ ± 1.42	61.57^e^ ± 1.52	51.59^de^ ± 1.64
	**400**	64.77^d^ ± 1.46	69.70^c^ ± 1.24	61.19^c^ ± 1.32
	**600**	71.85^b^ ± 2.08	76.69^b^ ± 1.23	67.13^b^ ± 0.94
	**800**	75.29^a^ ± 1.35	81.51^a^ ± 1.36	70.99^a^ ± 1.44
**LSD ≥ 0.05**		2.47	3.12	2.52

Electrostatic interactions between chitosan and its derivatives and bacterial cell surfaces alter cell walls (gram-positive) or outer membranes (gram-negative), disrupting membrane permeability and causing antibacterial effects. Chitosan and its derivatives also show enhanced antifungal properties at lower pH^53^. However, carboxymethyl chitosan (CCS) promotes cellular uptake and antibacterial efficacy due to enhanced positive charge and greater solubility, contributing to greater interaction with microbial cell surfaces. With MSE loading, CCS further enhances these effects by stabilizing and concentrating phenolic compounds, enabling membrane disruption via synergistic electrostatic/phenolic effects, and providing pH-responsive release.

### Antimicrobial activity by live/dead assay

**Fig. 3:**
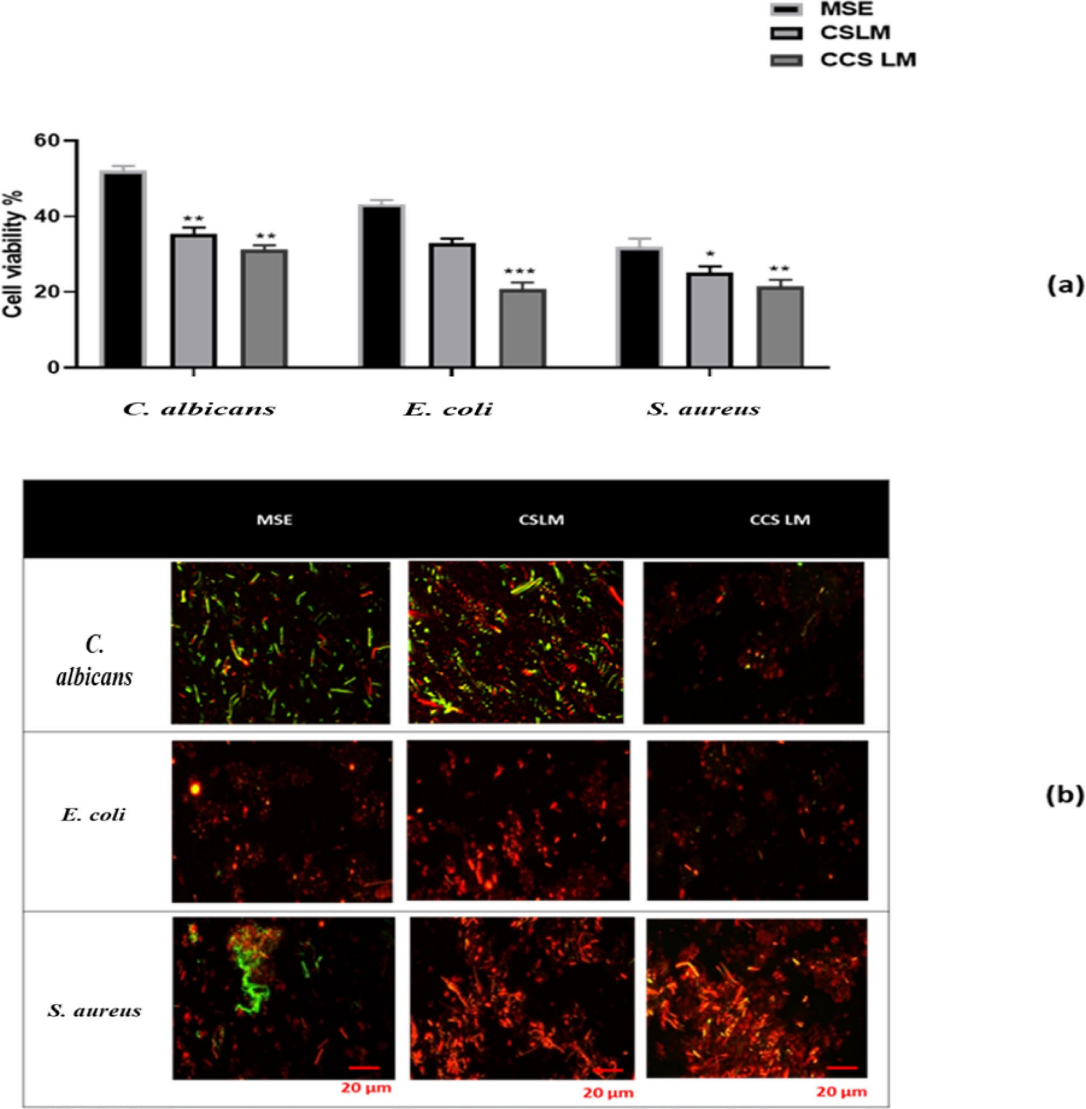
*Antimicrobial evaluation****.*** *(a) Cell viability (%) of C. albicans*,* E. coli*,* and S. aureus treated with MSE*,* CS LM*,* and CCS LM using the Live/Dead assay. (b) Confocal images showing green fluorescence for live and red for dead cells. Statistical significance: Viability reduction was significantly greater in CCS LM (p < 0.1234) than CS LM and MSE.*

The antimicrobial potential of the MSE and loaded extracts based on the live/dead assay against specific microbes (*C. albicans*, *E. coli*, and *S. aureus*) is presented in Fig. 3**(a) and (b)**. The loaded extracts with CS NPs and CCS NPs had significantly high antibacterial activity against microbes; for CS LM, the percentages of viable *C. albicans*, *E. coli*, and *S. aureus* cells were 36.35, 33.66, and 24.05%, respectively, whereas for CCS LM, the percentages of viable cells were 30.57, 21.81, and 22.56%, respectively. Finally, the activities of the loaded extracts (CS LM and CCS LM) were significantly greater than those of the MSE.

### Effect of edible coating on fruit quality parameters in strawberry fruits

**Fig. 4:**
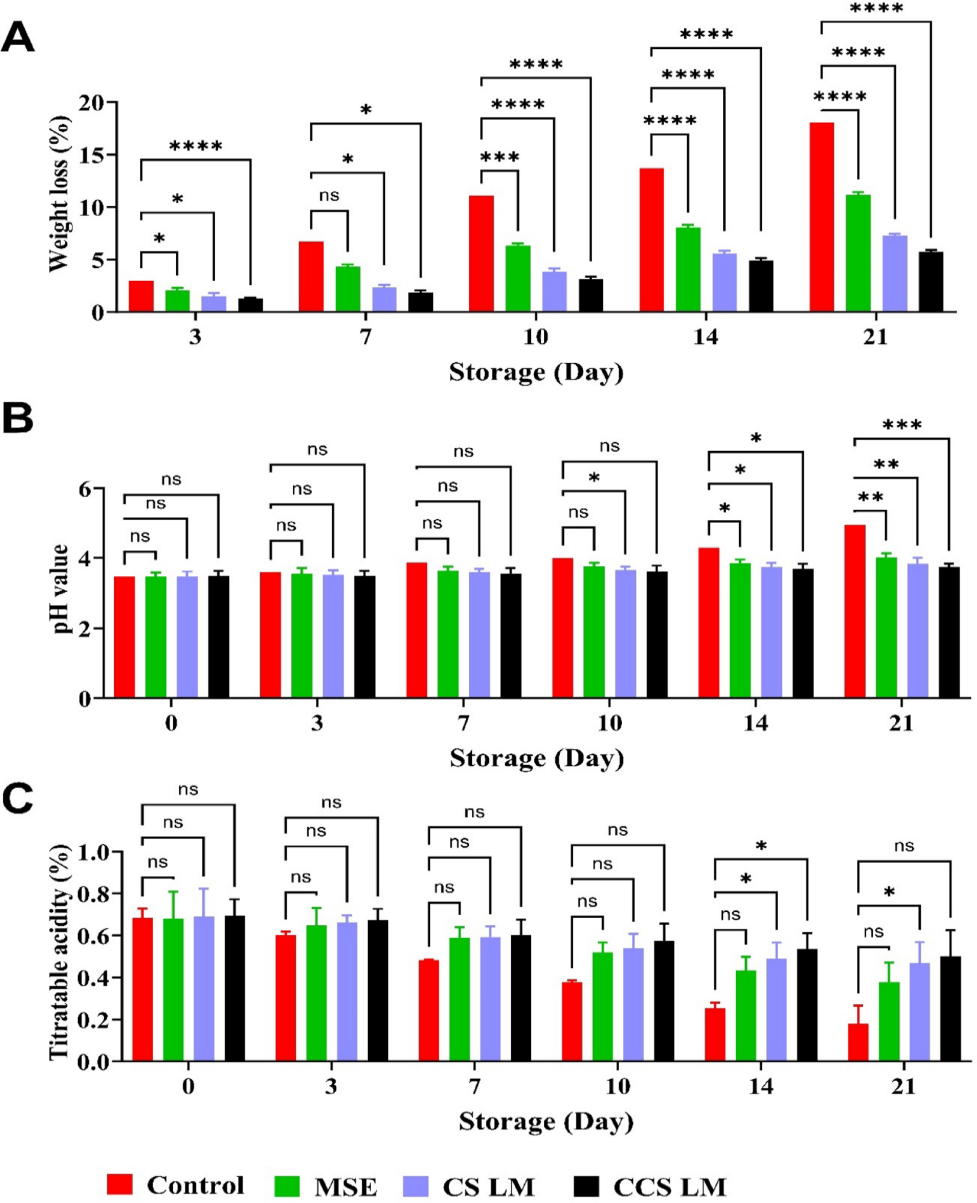
*Physicochemical quality parameters of strawberries during 21 days of storage at 4 °C****.*** *(a) Weight loss (%)*,* (b) pH changes*, (c) Titratable acidity (%). *Significance: CCS LM significantly reduced weight loss and pH changes while maintaining TA (***p < 0.002).

Fruit weight loss during cold storage of strawberries is primarily due to transpiration and respiration through the skin. Edible coatings provide a protective layer to reduce spoilage and microbial growth^54^. Weight loss was evaluated in uncoated fruits (control) and strawberries coated with MSE, CS LM, and CCS LM during 21 days of cold storage. Weight loss increased in all samples over time but was significantly less in coated versus uncoated strawberries (Fig. 4a). Fruits coated with CCS LM showed the lowest physiological weight loss (5.70%), followed by those coated with CS LM (7.26%) compared to strawberries coated with MSE alone (11.17%). Moreover, the control group lost the most physiological weight (18.06%) at the end of the storage period (21 days). Earlier works on strawberries coated with guava leaf-based chitosan nanoparticles (Gl-CSNPs) reported that weight loss was approximately 6.71% compared to that of uncoated fruits (25.37%) during cold storage for 12 days^55^.

The water retention capacity in carboxymethyl chitosan (CCS LM) is due to the carboxymethyl groups that enhance hydrophilicity and promote strong hydrogen bonding with water molecules. However, cross-linking CCS with mango seed extract (MSE) results in a dense, semi-permeable film that decreases hydrophilicity and water vapor transmission, thus forming a hydrophobic network that facilitates water retention. CCS LM is more effective than CS LM since its carboxymethyl groups retain water, inhibiting evaporation^56–58^.

Titratable acidity is correlated with the organic acid content in fruit and can decrease due to metabolic changes or organic acid consumption during respiration. The titratable acidity (TA) and pH of the coated and uncoated strawberries were measured during 21 days of cold storage (Fig. 4b-c). There was a significant decrease in TA in strawberry fruits after cold storage in all the samples during storage, but the coated fruits retained significantly better than the uncoated fruits. Strawberries coated with CCS LM showed the lowest TA reduction (0.50%, with loss 26.47%), followed by those coated with CS LM (0.46%, with loss 32.64%), compared to those coated with MSE alone (0.37%, with loss 44.41%), and while uncoated ones exhibited the most significant decrease (0.18%, with loss 74.11%). Previous studies have shown reduced titratable acidity (TA) in strawberries coated with chitosan incorporated with olive leaf or olive pomace extracts compared to uncoated strawberries^59^. Concurrently, the pH of the strawberries increased during storage for all the treatments, yielding significant differences between the coated and uncoated samples. It has been reported that increased oxygen levels in a fruit’s respiration rate may increase its pH during storage^60^. Among all the coating treatments, the pH values of strawberries coated with CCS LM and CS LM were the lowest (3.74 and 3.84), with no significant differences compared to those of the uncoated or MSE-coated fruits. A decrease in titratable acidity (TA) and an increase in pH are typical during fruit ripening and postharvest changes. Edible coatings as CCS LM can slow strawberry respiration, delaying the use of organic acids in enzymatic reactions during cold storage, and extending shelf life^38^. The modified structure of CCS LM, with reduced hydroxyl groups, may contribute to reduced interactions with organic acids, maintaining TA and pH by minimizing binding or reactions with these acids^61^.

**Fig. 5:**
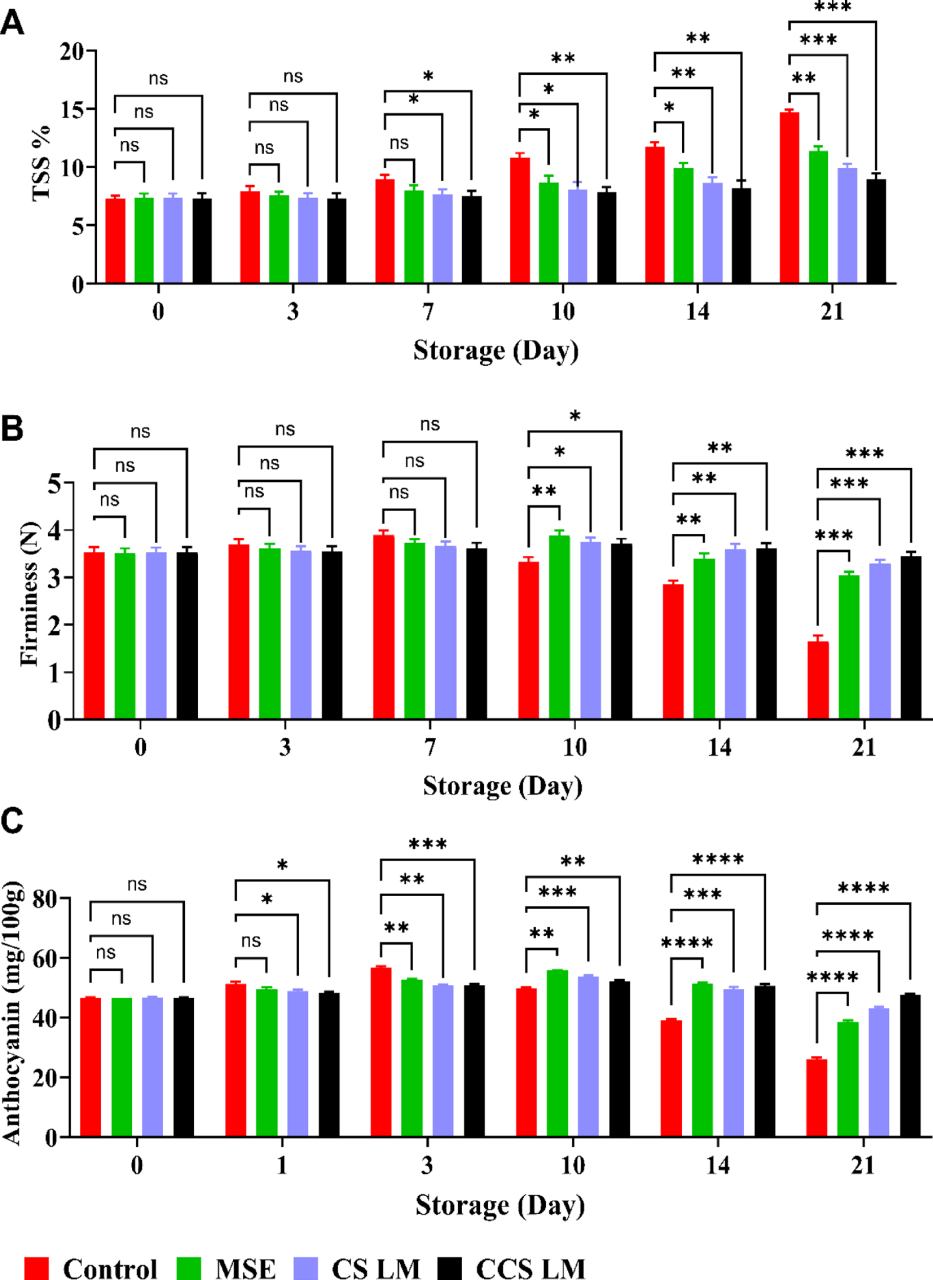
**(A)** *Additional fruit quality parameters.* *(a) Total soluble solids (% TSS)*,* (b) Firmness retention (%)*, (c) Anthocyanin content (mg/100 g). *Significance: CCS LM coatings resulted in significantly higher firmness and anthocyanin retention (**p < 0.021).

Total soluble solids (TSSs) strongly influence fruit quality and consumer acceptance. The TSS content of both uncoated and coated strawberry fruits during cold storage at 4 °C for 21 days is shown in Fig. 5a. However, after 21 days of cold storage at 4 °C, the TSS content was significantly greater in the control fruits (14.65%) than in the strawberries coated with CCS LM, CS LM, or MSE. This suggests that the uncoated fruit had active metabolic activity, converting starch into sugars and acids, thereby increasing soluble solids. Water loss and cell wall breakdown in mature strawberries also increased TSS in the control samples^42^. Among the coated strawberries, CCS LM showed the lowest increase in TSS (8.93%, an increase of 18.25%), followed by CS LM (9.9%, an increase of 25.65%), compared to MSE, which had a higher TSS of 11.36%, with increasing percent 50.03%. These results align with a previous study showing that chitosan coatings with olive extracts limited the increase in TSS in stored strawberries compared to that in uncoated fruit^59^.

It is important to note that coated strawberries’ minimal TSS increase reflects internal environment changes, possibly reducing O_2_ and increasing CO_2_. As indicated, the modified CCS and bioactive components in the mango seed extract reduce respiration rate and metabolic activity, including sugar conversion. This combination protects strawberry integrity, cell structure, and intracellular fluids^62^.

Firmness is crucial for fresh fruits, and the degradation of cell walls leads to a decrease in firmness over time. The coating materials significantly influenced strawberry firmness throughout the 21 days of cold storage at 4 °C (Fig. 5b). The firmness of the uncoated samples increased during the first 7 days, followed by a more rapid decrease until the end of storage (53.45%). Among the coatings, CCS LM and CS LM were more effective, with 2.64% and 6.62% decreases, respectively, compared to MSE-coated strawberries, which declined by 13.67%^56^. Previous studies reported that strawberries packaged with electrospun PVA/JE (jujube extract loaded into Poly vinyl alcohol) nanofiber retained better firmness compared to controls during 15 days of storage at 4 °C. Strawberry softening is caused by insoluble pectin and protopectin breakdown, which contribute to structural rigidity. Coating fruits with low oxygen and high carbon dioxide suppresses softening enzymes. Strawberry CCS LM and CS LM coatings retain firmness better than MSE coatings due to their hydrophilic characteristics, which prevent water evaporation and maintain turgidity. Bioactive mango seed extract components increase water loss prevention and MSE stability and efficiency^49,63^.

Remarkably, during the preliminary cold storage stage at 4 °C, no significant differences were detected in the anthocyanin content between the uncoated and coated strawberries at zero time (Fig. 5c). After 7 days of storage, the control group had the maximum anthocyanin concentration (56.58 mg/100 g), which then rapidly decreased to 25.89 mg/100 g, a 54.24% decrease at the end of storage period. Natural fruit ripening and significant weight loss cause pigment concentration^64^. Over the storage period, the decrease in anthocyanin levels in uncoated fruits might be related to the increasing activity of enzymes, such as polyphenol oxidase^65^.

Moreover, the anthocyanin content of the coated strawberry fruits continuously increased during the first 10 days of storage, reaching 52.12 to 55.75 mg/100 g. After 14 days, the losing percentage of the anthocyanin content to the coated strawberry with CCS LM and CS LM reached to 8.86 and 19.76%, respectively, compared to MSE-coated strawberry fruits, which declined to 31.04%. Similar behavior was also observed for cold-stored strawberries^66^, who reported that the anthocyanin content of strawberries coated with chitosan decreased the anthocyanin decay rate during strawberry storage compared to that of the uncoated samples. Anthocyanins indicate attractiveness and ripening stage in strawberries. Therefore, chitosan coatings, acting as gas barriers, modify the internal fruit atmosphere by increasing carbon dioxide (CO_2_) levels and reducing oxygen (O_2_) levels^67^, thereby inhibiting the enzymatic degradation of anthocyanins, rather than suppressing their synthesis. Compared with MSE, CCS LM exhibited the best performance among the different coating materials, followed by CS LM.

The edible coatings, particularly CCS LM and CS LM affect enzymatic browning, crucial in anthocyanin and color preservation. Browning in strawberries is catalyzed by PPO and POD by oxidizing phenolic compounds to quinones, resulting in color loss. Coatings suppress oxygen (O₂) and promote carbon dioxide (CO₂) in fruits, preventing PPO and POD activity, slowing browning, and preserving anthocyanin. Additionally, mango seed extract (MSE) antioxidant properties improve anthocyanin retention by inhibiting free radicals and oxidative stress. This double mechanism (antioxidant + gas barrier) enhances anthocyanin stability during storage^68^.

**Fig. 6:**
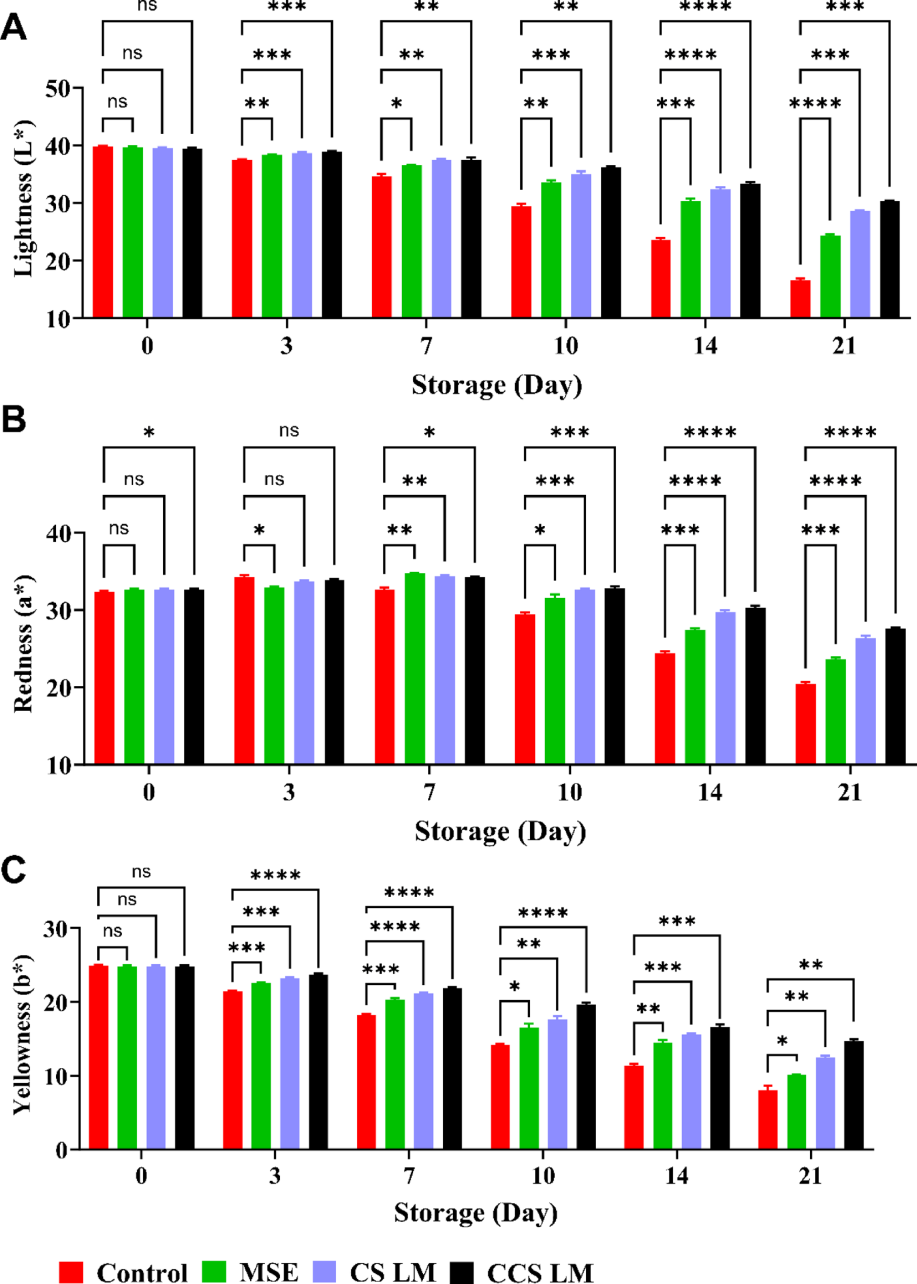
*Color attributes of strawberries.* Changes in (a) L* (lightness), (b) a* (redness), and (c) b* (yellowness) of uncoated and coated strawberries with MSE,* CS LM*, and CCS LM at 4 °C for 21 days. *Significance: CCS LM and CS LM significantly delayed color degradation (*p < 0.0332).

Fruit color, measured by L* (lightness), a* (redness), and b* (yellowness) values, is important for assessing strawberry quality during storage. Both coated and uncoated strawberries showed decreasing L*, a*, and b* values over the 21-day storage period, indicating fruit darkening and color deterioration (Fig. 6). However, uncoated fruits exhibited the most dramatic L* and b* reductions (58.39 and 67.63%, respectively), reaching the darkest and least yellow color. Uncoated strawberries also showed an initial increase in a* through 3 days, followed by a substantial decrease of 40.41% by day 21. The decrease in L*, a*, and b* values in uncoated strawberries can be attributed to various factors, including oxidative browning, moisture loss leading to fruit shriveling and reduced light reflection, enzymatic activity degrading pigments, and microbial activity accelerating anthocyanin degradation.

Among the various coating materials, strawberries coated with CCS LM and CS LM exhibited the lowest decreases in L* and b* (23.10% and 40.89%, respectively, for CCS LM; 27.66% and 49.84% for CS LM, respectively, compared to MSE alone (38.70% and 59.34%, respectively). After 7 days of storage, the coated strawberries showed an increase in a* values, with CCS LM (19.28%) and CS LM (23.32%) showing the most negligible deterioration compared to MSE (31.90%). Similar results have been reported by Siburian et al.^69^., who noted that compared with uncoated samples, strawberries coated with alginate enriched with cinnamon essential oil exhibited a decrease in the L*, a*, and b* values after 12 days of cold storage. The coating materials helped retain the natural red color of the strawberries by preventing enzymatic oxidation and browning reactions that degrade fruit pigments^70^. Compared with MSE alone, CCS LM was most effective at maintaining L*, a*, and b* values, which can be attributed to modifications in the CCS structure and increased stability of mango seed extracts when incorporated into the CCS coating.

#### **The microbial count of strawberries coated with CCS LM**,** CS LM**,** MSE**,** and uncoated samples during storage at 4 °C**

**Fig. 7:**
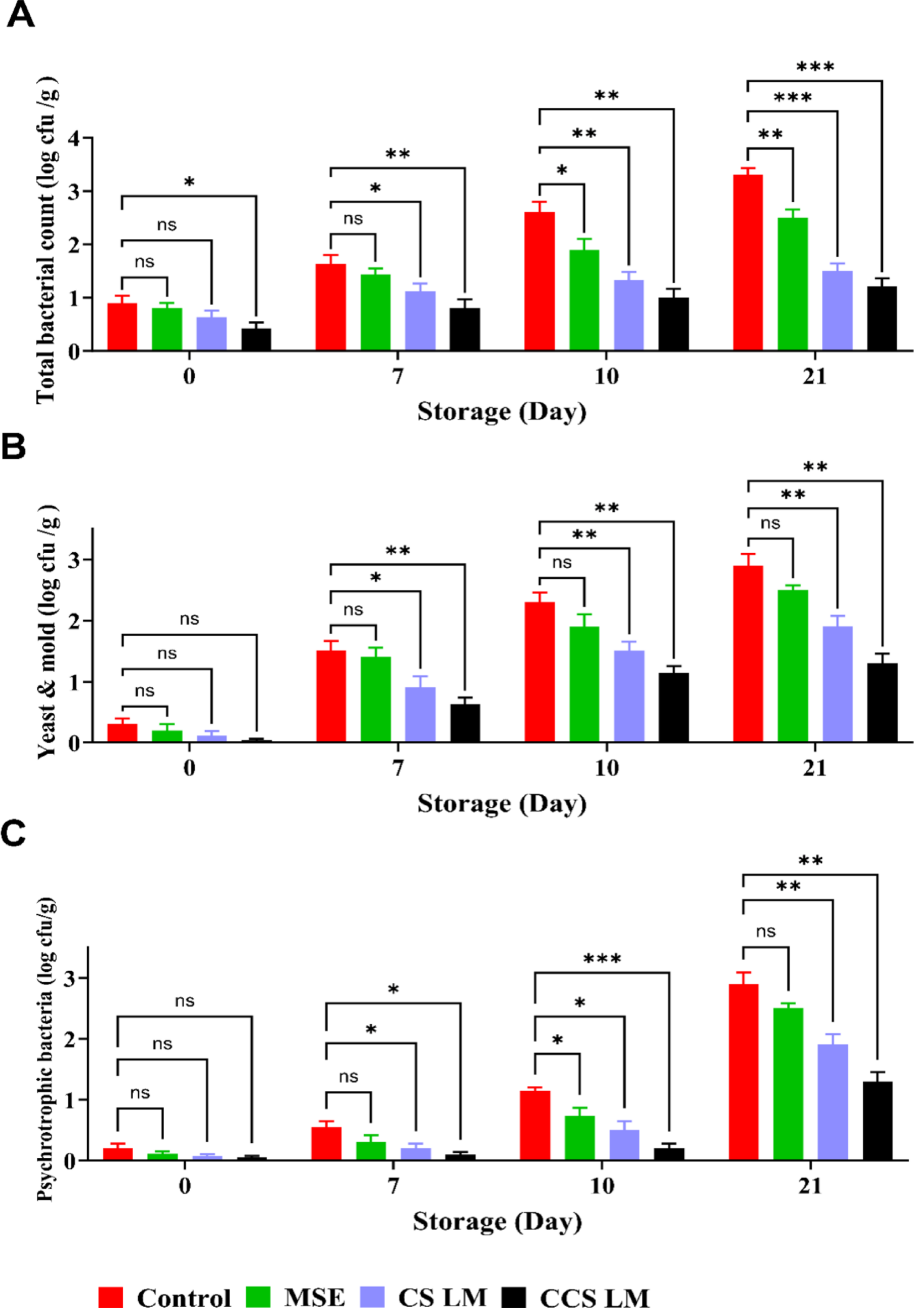
*Total microbial load of strawberries coated with MSE, CS LM, and CCS LM during 21 days of cold storage at 4 °C.* *(A) Total bacterial count*,* (B) yeast and mold count*,* and (C) Psychrotrophic bacterial count (log₁₀ CFU/g). CCS LM-coated strawberries exhibited the lowest microbial growth compared to CS LM*,* MSE*,* and control samples. Values are means ± standard error (n = 3). Different letters indicate statistically significant differences (p < 0.1234).*

**Fig. 8:**
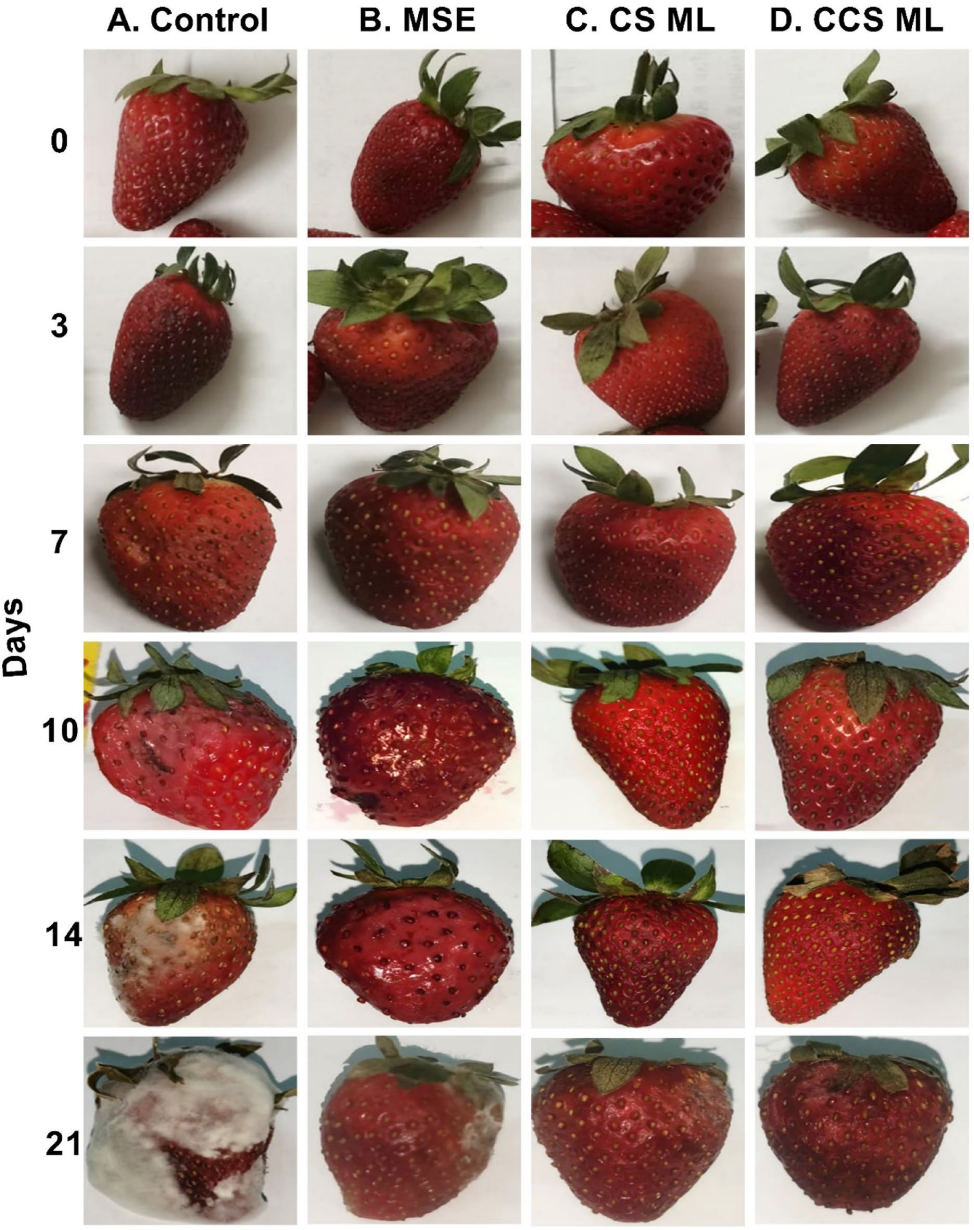
*Visual assessment of strawberries after 21 days of storage (A) Control, (B) MSE (C) CS LM*,* and (D) CCS LM showing physical deterioration and preservation.*

The population showed variation due to treatment effects during storage (Figs. 7 and 8). Overall, compared with MSE, CCS LM had the lowest psychrotrophic bacteria count and total bacterial count (0.31 ± 0.12 and 1.2 ± 0.15 log_10_ CFU/gm, respectively), followed by CS LM (0.63 ± 0.14 and 1.5 ± 0.3 log_10_ CFU/gm, respectively). In contrast, the maximal psychrotrophic bacteria count in the control sample was 1.4 ± 0.13 and 3.3 ± 0.15 log_10_ CFU/g in the control (uncoated) sample after 21 days. On the other hand, strawberries coated with CCS LM had the lowest fungi count (1.3 log_10_ CFU/g) after 21 days compared to MSE. Moreover, the control’s maximal fungal count was 2.3 ± 0.08 log_10_ CFU/g.

Strawberries are spoiled at 6–7 log_10_ CFU/g, as this level is associated with visible mold, off-odors, and texture breakdown. After 21 days, the uncoated strawberry reached 3.3 log_10_ CFU/g with notable microbial growth but without reaching the spoilage threshold. CCS LM and CS LM-coated strawberries exhibited appreciably low microbial counts (1.2 log_10_ CFU/g and 1.5 log_10_ CFU/g), demonstrating their effectiveness in delaying spoilage^71^.

The 21-day storage time was utilized to evaluate the long-term effect of edible coatings (CCS LM, CS LM, and MSE) on strawberry quality in preserving strawberry quality under realistic storage conditions. Strawberries are highly perishable, with a typical shelf life of 7–10 days when refrigerated. For up to 21 days, we aimed to simulate long-distance transportation or extended storage, which are common challenges in the supply chain.

Notable microbial counts were linked to glucose percentages and protein levels. The presence of such nutrients can enhance the growth of microbes, with glucose acting as a primary energy source and proteins enabling microbial metabolism. Earlier research has also reported the same associations between nutritional value and microbial growth in strawberries. The antimicrobial activity of CCS LM and CS LM is due to the positive amine groups of chitosan and carboxymethyl chitosan, which interact with microbial cell membranes, disrupting membrane integrity and leading to cell lysis^72^. In addition, the carboxymethyl groups of CCS improve the charge and solubility of CCS, enabling it to penetrate microbial cells and inhibit growth. CCS LM had a lower microbial count than CS LM and MSE. Additionally, mango seed extract (MSE) has antimicrobial properties due to bioactive compound content, which are less effective than CCS LM or CS LM because they lack chitosan’s structural integrity and barrier function^24^.

## Conclusion

This work shows that chitosan (CS) and carboxymethyl chitosan (CCS) nanoparticles could be good carriers for mango seed extract (MSE) to make it more durable, antioxidant, and antibacterial. Encapsulating MSE in CS and CCS nanoparticles greatly increased how well phenolic compounds stayed stable in different storage settings. CCS LM was the most stable at all temperatures studied. Both CS LM and CCS LM showed strong inhibitory effects against *Staphylococcus aureus*,* Escherichia coli*, and *Candida albicans* in antimicrobial tests. CCS LM had the highest inhibition rates. Also, when used as edible coatings, CS LM and CCS LM greatly helped keep strawberries fresh after they were picked by lowering weight loss, keeping them firm, delaying changes in pH and titratable acidity, and slowing down the growth of bacteria. CCS LM consistently did better than the other formulations in all tests. These results show that CCS LM could be a good choice for a long-lasting, biodegradable, and useful coating for keeping fresh fruit fresh. Using plant-based bioactives and food-grade biopolymers instead of synthetic preservatives is better for the environment and helps food stay fresh longer and reduces food waste. Future studies would have to focus on augmenting manufacturing, analyzing sensory characteristics, and examining regulatory adherence for commercial utilization. The incorporation of nanocarrier-based edible coatings into postharvest systems may be an effective approach to enhance food quality and safety within fresh fruit supply chains.

## Data Availability

The data used during the current study available from the corresponding author on reasonable request.

## References

[1] Skrovankova, S., Sumczynski, D., Mlcek, J., Jurikova, T. & Sochor, J. Bioactive compounds and antioxidant activity in different types of berries. *Int. J. Mol. Sci.* **16**, 24673–24706. 10.3390/ijms161024673 (2015). 26501271 10.3390/ijms161024673 PMC4632771

[2] Sadik, H. et al. Comparison of the nutritional proprieties of commercial strawberries, red and black raspberry consumed in Morocco. *Appl. Food Res.* **3**, 100362. 10.1016/j.afres.2023.100362 (2023).

[3] Qureshi Quarshi, H. et al. Post-Harvest Problems of Strawberry and Their Solutions, in: Recent Stud. Strawberries, IntechOpen, (2023). 10.5772/intechopen.102963

[4] Satitmunnaithum, J. et al. Microbial population size and strawberry fruit firmness after drop shock-induced mechanical damage. *Postharvest Biol. Technol.* **192**, 112008. 10.1016/j.postharvbio.2022.112008 (2022).

[5] Damdam, A., Al-Zahrani, A., Salah, L. & Salama, K. N. Effect of combining UV‐C irradiation and vacuum sealing on the shelf life of fresh strawberries and tomatoes. *J. Food Sci.* **88**, 595–607. 10.1111/1750-3841.16444 (2023). 36624610 10.1111/1750-3841.16444 PMC10108318

[6] Perez-Vazquez, A., Barciela, P., Carpena, M. & Prieto, M. Edible coatings as a natural packaging system to improve fruit and vegetable shelf life and quality. *Foods* **12**, 3570. 10.3390/foods12193570 (2023). 37835222 10.3390/foods12193570 PMC10572534

[7] Priya, K., Thirunavookarasu, N. & Chidanand, D. V. Recent advances in edible coating of food products and its legislations: A review. *J. Agric. Food Res.* **12**, 100623. 10.1016/j.jafr.2023.100623 (2023).

[8] Ibrahim, H. M. et al. Efficacy improvement of tri-serotypes vaccine for Salmonella using nanomaterial-based adjuvant in chicken, Beni-Suef univ. *J. Basic. Appl. Sci.* **13**10.1186/s43088-024-00477-x (2024).

[9] Xie, Q., Luo, D., Mu, K. & Xue, W. Preparation and characterization of carboxymethyl chitosan/nano-MgO/red cabbage anthocyanins multifunctional films via colloid formation and its application on shrimp preservation, food Packag. *Shelf Life*. **37**, 101074. 10.1016/j.fpsl.2023.101074 (2023).

[10] Feng, Z. et al. Anti-Aspergillus Niger mechanism of small molecular combinations of essential oils and their application in extending the shelf-life of bread. *Food Biosci.* **64**, 105979. 10.1016/j.fbio.2025.105979 (2025).

[11] Feng, Z., Shao, B., Yang, Q., Diao, Y. & Ju, J. The force of Zein self-assembled nanoparticles and the application of functional materials in food preservation. *Food Chem.* **463**, 141197. 10.1016/j.foodchem.2024.141197 (2025). 39276690 10.1016/j.foodchem.2024.141197

[12] Feng, Z., Sun, P., Zhao, F., Li, M. & Ju, J. Advancements and challenges in biomimetic materials for food preservation: A review. *Food Chem.* **463**, 141119. 10.1016/j.foodchem.2024.141119 (2025). 39241425 10.1016/j.foodchem.2024.141119

[13] Gao, Q. et al. Application of nano-ZnO in the food preservation industry: antibacterial mechanisms, influencing factors, intelligent packaging, preservation film and safety. *Crit. Rev. Food Sci. Nutr.* 1–27. 10.1080/10408398.2024.2387327 (2024). 10.1080/10408398.2024.2387327 39097753

[14] Wang, J., Gao, Q., Zhao, F. & Ju, J. Repair mechanism and application of self-healing materials for food preservation. *Crit. Rev. Food Sci. Nutr.* **64**, 11113–11123. 10.1080/10408398.2023.2232877 (2024). 37427571 10.1080/10408398.2023.2232877

[15] Yang, P. et al. Synergistic anti-biofilm strategy based on essential oils and its application in the food industry. *World J. Microbiol. Biotechnol.* **41**, 81. 10.1007/s11274-025-04289-8 (2025). 40011295 10.1007/s11274-025-04289-8

[16] Martins, V. F. R., Pintado, M. E., Morais, R. M. S. C. & Morais, A. M. M. B. Valorisation of micro/nanoencapsulated bioactive compounds from plant sources for food applications towards sustainability. *Foods* **12**10.3390/foods12010032 (2023). 10.3390/foods12010032 PMC9818261 36613248

[17] Ncama, K., Magwaza, L. S., Mditshwa, A. & Tesfay, S. Z. Plant-based edible coatings for managing postharvest quality of fresh horticultural produce: A review. *Food Packag Shelf Life*. **16**, 157–167. 10.1016/j.fpsl.2018.03.011 (2018).

[18] Antunes, J. C. et al. Bioactivity of chitosan-based particles loaded with plant-derived extracts for biomedical applications: emphasis on antimicrobial fiber-based systems. *Mar. Drugs*. **19**10.3390/md19070359 (2021). 10.3390/md19070359 PMC8303307 34201803

[19] Popescu, P. A. et al. Chitosan-Based edible coatings containing essential oils to preserve the shelf life and postharvest quality parameters of organic strawberries and apples during cold storage. *Foods* **11**10.3390/foods11213317 (2022). 10.3390/foods11213317 PMC9657762 36359930

[20] Pavinatto, A. et al. Coating with chitosan-based edible films for mechanical/biological protection of strawberries. *Int. J. Biol. Macromol.* **151**, 1004–1011. 10.1016/j.ijbiomac.2019.11.076 (2020). 31726134 10.1016/j.ijbiomac.2019.11.076

[21] Sadh, P. K., Duhan, S. & Duhan, J. S. Agro-industrial wastes and their utilization using solid state fermentation: a review. *Bioresour Bioprocess.* **5**, 1–15. 10.1186/s40643-017-0187-z (2018).

[22] Thakur, J. & Borah, A. Microcapsules of bioactive compounds from fruits and vegetables waste and their utilization: A review. *Pharma Innov.* **10**, 151–157. 10.22271/tpi.2021.v10.i5c.6191 (2021).

[23] Arah, I. K., Ahorbo, G. K., Anku, E. K., Kumah, E. K. & Amaglo, H. Postharvest handling practices and treatment methods for tomato handlers in developing countries: A mini review. *Adv. Agric.* **2016**10.1155/2016/6436945 (2016).

[24] Ch Momin, M., Jamir, A. R., Ankalagi, N., Bijaya Devi, O. & Henny, T. Edible coatings in fruits and vegetables: A brief review, ~ 71 ~ pharma. *Innov. J.* **10**, 71–78 (2021). http://www.thepharmajournal.com

[25] Bernal-Mercado, A. T. et al. Javier Vazquez-Armenta, antioxidant and antimicrobial capacity of phenolic compounds of Mango (Mangifera indica L.) seed depending upon the extraction process. *J. Med. Plants By-Products*. **2**, 209–219. 10.22092/jmpb.2018.118149 (2018).

[26] El, A. M. & Anany Nutritional composition, antinutritional factors, bioactive compounds and antioxidant activity of guava seeds (Psidium Myrtaceae) as affected by roasting processes. *J. Food Sci. Technol.* **52**, 2175–2183. 10.1007/s13197-013-1242-1 (2015). 25829598 10.1007/s13197-013-1242-1 PMC4375223

[27] Wolfe, K., Wu, X. & Liu, R. H. Antioxidant activity of Apple peels. *J. Agric. Food Chem.* **51**, 609–614. 10.1021/jf020782a (2003). 12537430 10.1021/jf020782a

[28] Zhishen, J., Mengcheng, T. & Jianming, W. The determination of flavonoid contents in mulberry and their scavenging effects on superoxide radicals. *Food Chem.* **64**, 555–559. 10.1016/S0308-8146(98)00102-2 (1999).

[29] Abd El-Aziz, W. R. et al. Evaluation of cell-mediated immunity of e.coli nanovaccines in chickens. *J. Immunol. Methods*. **506**, 1–9. 10.1016/j.jim.2022.113280 (2022). 10.1016/j.jim.2022.113280 35577101

[30] Kostadinova, A. et al. Interactions of Chitosan-Based nanoparticles with Bio-Inspired membranes. *Oxid. Commun.* **71**, 63–71 (2021).

[31] Mohamed, S. Y., Elshoky, H. A., El-Sayed, N. M., Fahmy, H. M. & Ali, M. A. Ameliorative effect of zinc oxide-chitosan conjugates on the anticancer activity of cisplatin: approach for breast cancer treatment. *Int. J. Biol. Macromol.* **257**, 128597. 10.1016/j.ijbiomac.2023.128597 (2024). 38056740 10.1016/j.ijbiomac.2023.128597

[32] Elshoky, H. A., Salaheldin, T. A., Ali, M. A. & Gaber, M. H. Ascorbic acid prevents cellular uptake and improves biocompatibility of Chitosan nanoparticles. *Int. J. Biol. Macromol.* **115**, 358–366. 10.1016/j.ijbiomac.2018.04.055 (2018). 29653170 10.1016/j.ijbiomac.2018.04.055

[33] Cabral, B. R. P. et al. Improving stability of antioxidant compounds from Plinia cauliflora (jabuticaba) fruit Peel extract by encapsulation in Chitosan microparticles. *J. Food Eng.* **238**, 195–201. 10.1016/j.jfoodeng.2018.06.004 (2018).

[34] Re, R. et al. Antioxidant activity applying an improved ABTS radical cation decolorization assay, free Radic. *Biol. Med.* **26**, 1231–1237. 10.1016/S0891-5849(98)00315-3 (1999). 10.1016/s0891-5849(98)00315-3 10381194

[35] Brand-Williams, W., Cuvelier, M. E. & Berset, C. Use of a free radical method to evaluate antioxidant activity. *LWT - Food Sci. Technol.* **28**, 25–30. 10.1016/S0023-6438(95)80008-5 (1995).

[36] Kalemba, D. & Kunicka, A. Antibacterial and antifungal properties of essential oils. *Curr. Med. Chem.* **10**, 813–829. 10.2174/0929867033457719 (2005). 10.2174/0929867033457719 12678685

[37] Ibrahim, H. M. et al. Polymeric nanocarrier-based adjuvants to enhance a locally produced mucosal Coryza vaccine in chicken. *Sci. Rep.* **14**, 15262. 10.1038/s41598-024-65267-y (2024). 38961116 10.1038/s41598-024-65267-y PMC11222434

[38] Hern, P. Effect of calcium dips and Chitosan coatings on postharvest life of strawberries (Fragaria x ananassa), 39 247–253. (2006). 10.1016/j.postharvbio.2005.11.006

[39] Guerreiro, A. C., Gago, C. M. L., Faleiro, M. L., Miguel, M. G. C. & Antunes, M. D. C. The use of polysaccharide-based edible coatings enriched with essential oils to improve shelf-life of strawberries. *Postharvest Biol. Technol.* **110**, 51–60. 10.1016/j.postharvbio.2015.06.019 (2015).

[40] Côté, J. et al. Effects of juice processing on cranberry antioxidant properties. *FRIN* **44**, 2907–2914. 10.1016/j.foodres.2011.06.052 (2011).

[41] Wehr, H. M. & Frank, J. F. Standard methods for the examination of dairy products. *Am. Public. Health Association*. 10.2105/9780875530024 (2004).

[42] Duan, J., Wu, R., Strik, B. C. & Zhao, Y. Effect of edible coatings on the quality of fresh blueberries (Duke and Elliott) under commercial storage conditions. *Postharvest Biol. Technol.* **59**, 71–79. 10.1016/j.postharvbio.2010.08.006 (2011).

[43] Nn, A. A review on the extraction methods use in medicinal plants, principle, strength and limitation. *Med. Aromat. Plants*. **04**, 3–8. 10.4172/2167-0412.1000196 (2015).

[44] El-Kady, T., Abd El-Rahman, M. & A.O.Toliba, A. O. T. Abo El-maty, evaluation of Mango seed kernel extract as natural occurring phenolic rich antioxidant compound. *Bull. Natl. Nutr. Inst.* **48**, 1–30. 10.21608/bnni.2017.4239 (2017).

[45] El-Sayed, N. M. et al. Synthesis and characterization of mussel-inspired nanocomposites based on dopamine–chitosan–iron oxide for wound healing: in vitro study. *Int. J. Pharm.* **632**, 122538. 10.1016/j.ijpharm.2022.122538 (2023). 36586630 10.1016/j.ijpharm.2022.122538

[46] Ghaderi Ghahfarokhi, M., Barzegar, M., Sahari, M. A. & Azizi, M. H. Enhancement of thermal stability and antioxidant activity of thyme essential oil by encapsulation in Chitosan nanoparticles. *J. Agric. Sci. Technol.* **18**, 1781–1792. https://doi.org/DOR:20.1001.1.16807073.2016.18.7.20.0 (2016).

[47] Soleymanfallah, S., Khoshkhoo, Z., Hosseini, S. E. & Azizi, M. H. Preparation, physical properties, and evaluation of antioxidant capacity of aqueous grape extract loaded in chitosan-TPP nanoparticles. *Food Sci. Nutr.* **10**, 3272–3281. 10.1002/fsn3.2891 (2022). 36249981 10.1002/fsn3.2891 PMC9548353

[48] Wang, L. et al. Preparation and antioxidant activity of new carboxymethyl Chitosan derivatives bearing Quinoline groups. *Mar. Drugs*. **21**10.3390/md21120606 (2023). 10.3390/md21120606 PMC10745101 38132927

[49] Bian, L., Sun, H., Zhou, Y., Tao, Y. & Zhang, C. Enhancement of antioxidant property of N-Carboxymethyl Chitosan and its application in strawberry preservation. *Molecules* **27**10.3390/molecules27238496 (2022). 10.3390/molecules27238496 PMC9735828 36500590

[50] Wang, Z. et al. Effect of Chitosan and its Water-Soluble derivatives on antioxidant activity, polymers (Basel). **16** 867. (2024). 10.3390/polym16070867 10.3390/polym16070867 PMC11013083 38611124

[51] de Brito, G. O. et al. Phenolic compound profile by UPLC-MS/MS and encapsulation with Chitosan of spondias Mombin L. Fruit Peel extract from Cerrado Hotspot—Brazil. *Molecules* **27**10.3390/molecules27082382 (2022). 10.3390/molecules27082382 PMC9028924 35458580

[52] Gradinaru, L. M. et al. Chitosan membranes containing plant extracts: preparation, characterization and antimicrobial properties. *Int. J. Mol. Sci.* **24**, 8673. 10.3390/ijms24108673 (2023). 37240023 10.3390/ijms24108673 PMC10217953

[53] Confederat, L. G., Tuchilus, C. G., Dragan, M., Sha’at, M. & Dragostin, O. M. Preparation and antimicrobial activity of Chitosan and its derivatives: A concise review. *Molecules* **26**, 3694. 10.3390/molecules26123694 (2021). 34204251 10.3390/molecules26123694 PMC8233993

[54] Lufu, R., Ambaw, A. & Opara, U. L. The contribution of transpiration and respiration processes in the mass loss of pomegranate fruit (cv. Wonderful). *Postharvest Biol. Technol.* **157**, 110982. 10.1016/j.postharvbio.2019.110982 (2019).

[55] Ali, L. M., E.R.A.E, A., Hasan, H. E. S., Suliman, A. E. R. E. & Saleh, S. S. Quality characteristics of strawberry fruit following a combined treatment of laser sterilization and guava leaf-based Chitosan nanoparticle coating. *Chem. Biol. Technol. Agric.* **9**, 1–13. 10.1186/s40538-022-00343-x (2022).

[56] Riaz, A. et al. Application of chitosan-based Apple Peel polyphenols edible coating on the preservation of strawberry (Fragaria Ananassa Cv Hongyan) fruit. *J. Food Process. Preserv*. **45**10.1111/jfpp.15018 (2021).

[57] Chaiwong, N. et al. Synergistics of carboxymethyl Chitosan and mangosteen extract as enhancing moisturizing, antioxidant, antibacterial, and deodorizing properties in emulsion cream. *Polym. (Basel)*. **14**, 178. 10.3390/polym14010178 (2022). 10.3390/polym14010178 PMC8747190 35012200

[58] Wu, Y. et al. Effect of different crosslinking agents on carboxymethyl chitosan-glycyrrhizic acid hydrogel: characterization and biological activities comparison. *Int. J. Biol. Macromol.* **298**, 139977. 10.1016/j.ijbiomac.2025.139977 (2025). 39826743 10.1016/j.ijbiomac.2025.139977

[59] Khalifa, I., Barakat, H., El-Mansy, H. A. & Soliman, S. A. Effect of Chitosan–Olive oil processing residues coatings on keeping quality of Cold-Storage strawberry (Fragaria ananassa. Var. Festival). *J. Food Qual.* **39**, 504–515. 10.1111/jfq.12213 (2016).

[60] Dong, Y. et al. Elevated oxygen contributes to the promotion of polyphenol biosynthesis and antioxidant capacity: A case study on strawberries. *Horticulturae* **11**, 107. 10.3390/horticulturae11010107 (2025).

[61] Yang, C. et al. Carboxymethyl Chitosan coated alpha-linolenic acid nanoliposomes: preparation, stability and release in vitro and in vivo. *Food Chem.* **404**, 134526. 10.1016/j.foodchem.2022.134526 (2023). 36265276 10.1016/j.foodchem.2022.134526

[62] Quintana, S. E., Llalla, O., García-Risco, M. R. & Fornari, T. Comparison between essential oils and supercritical extracts into chitosan-based edible coatings on strawberry quality during cold storage. *J. Supercrit Fluids*. **171**, 105198. 10.1016/j.supflu.2021.105198 (2021).

[63] Mshora, A., Gill, D. P., Jawandha, D. S., Sinha, A. & Singh, D. M. Effect of Chitosan coatings on physico-chemical and enzymatic activities in Mango Cv Dashehari stored at low temperature. *J. Hortic. Sci.* **17**, 381–387. 10.24154/jhs.v17i2.1015 (2022).

[64] Sogvar, O. B., Koushesh Saba, M. & Emamifar, A. Aloe Vera and ascorbic acid coatings maintain postharvest quality and reduce microbial load of strawberry fruit. *Postharvest Biol. Technol.* **114**, 29–35. 10.1016/j.postharvbio.2015.11.019 (2016).

[65] Estrada-Girón, Y., Cabrera-Díaz, E., Esparza-Merino, R. M., Martín-del-Campo, A. & Valencia-Botín, A. J. Innovative edible films and coatings based on red color pectin obtained from the byproducts of hibiscus Sabdariffa L. for strawberry preservation. *J. Food Meas. Charact.* **14**, 3371–3380. 10.1007/s11694-020-00577-z (2020).

[66] Taha, I., Shahat, M., Mohamed, M. & Osheba, A. Improving the quality and shelf-life of strawberries as coated with nano-edible films during storage. *Al-Azhar J. Agric. Res.* **45**, 1–14. 10.21608/ajar.2020.149403 (2020).

[67] Meio, N. F. C. B. et al. Quality of postharvest strawberries: comparative effect of fungal Chitosan gel, nanoparticles and gel enriched with edible nanoparticle coatings. *Int. J. Food Stud.* **9**, 373–393. 10.7455/ijfs/9.2.2020.a9 (2020).

[68] Adiletta, G., Di Matteo, M. & Petriccione, M. Multifunctional role of Chitosan edible coatings on antioxidant systems in fruit crops: A review. *Int. J. Mol. Sci.* **22**10.3390/ijms22052633 (2021). 10.3390/ijms22052633 PMC7961546 33807862

[69] Siburian, P. W., Falah, M. A. F. & Mangunwikarta, J. Alginate-based edible coatings enriched with cinnamon essential oil extend storability and maintain the quality of strawberries under tropical condition. *PLANTA Trop.* **9**, 58–70. 10.18196/pt.v9i1.10368 (2021).

[70] De Bruno, A., Gattuso, A., Ritorto, D., Piscopo, A. & Poiana, M. Effect of edible coating enriched with natural antioxidant extract and Bergamot essential oil on the shelf life of strawberries. *Foods* **12**10.3390/foods12030488 (2023). 10.3390/foods12030488 PMC9914418 36766017

[71] Drobek, M. et al. The use of interactions between microorganisms in strawberry cultivation (Fragaria x Ananassa Duch.)., Front. *Plant. Sci.* **12**, 780099. 10.3389/fpls.2021.780099 (2021). 10.3389/fpls.2021.780099 PMC8668414 34917112

[72] Nadarajah, K. *Development and Characterization of Antimicrobial Edible Films from Crawfish Chitosan* (Louisiana State University and Agricultural & Mechanical College, 2005).

