# Correction: Chitosan and carboxymethyl chitosan nanocarriers enhance mango seed extract stability and antimicrobial activity to improve strawberry postharvest quality

**DOI:** 10.1038/s41598-026-35869-9

**Published:** 2026-01-22

**Authors:** Eman S. El-Ashaal, Hisham A. Elshoky, Nayera M. El-Sayed, Ebtehal A. El-Kholany

**Affiliations:** 1 Nanotechnology and Advanced Material Central Lab, Agricultural Research Center, Giza, 12619 Egypt; 2 Regional Center for Food and Feed, Agricultural Research Center, Giza, 12619 Egypt; 3 Physics Department, Faculty of Science, Mansoura University, Mansoura, 35516 Egypt; 4 Special Food and Nutrition Department, Food Technology Research Institute, Agricultural Research Center, Giza, 12619 Egypt

Correction to: *Scientific Reports* 10.1038/s41598-025-16756-1, published online 26 August 2025

The original version of this Article contained an error in the name of author Ebtehal A. El-Kholany, which was incorrectly given as Ebtehal A. El-Kholony.

The original Article has been corrected.

